# Hotspot Interactions between Two Fab Molecules in Molecular Dynamics Simulations Improve Predictive Models of Aggregation Kinetics

**DOI:** 10.1021/acs.molpharmaceut.5c01464

**Published:** 2026-02-09

**Authors:** Yuhan Wang, Hywel D. Williams, Duygu Dikicioglu, Paul A. Dalby

**Affiliations:** † Department of Biochemical Engineering, 4919 University College London, London Wc1e 6bt, U.K.; ‡ 493931 CSL Ltd, Biopharmaceutical Product Development, 655 Elizabeth Street, Melbourne, VIC 3000 Australia

**Keywords:** antibody aggregation, protein−protein interaction (PPI), molecular dynamics simulations, model building, multiple antibody fragments, hotspot regions

## Abstract

Protein–protein interactions (PPIs) are fundamental to numerous biological processes, and the identification of interaction hotspots is essential for understanding the mechanisms of protein aggregation and informing protein engineering efforts. Although various algorithms have been developed to predict hotspot regions for protein–protein interactions, little research has focused on understanding the relative roles of these largely protein surface-based interactions and the interactions between cross-β sheet-forming aggregation-prone regions (APRs) that are largely buried within proteins. This study uses all-atom molecular dynamics (MD) simulations to investigate the interactions between two Fab antibody fragments, focusing on the identification and characterization of the key interaction sites. Through frequency contact map analysis and principal component analysis, we identified specific residues that consistently formed stable contacts, distinguishing them from transient random interactions. Our findings revealed that while numerous contact points occurred throughout the simulations, relatively few sites acted as persistent hotspots based on the duration of their contacts. Comparison to single Fab simulations highlighted the influence of interfragment interactions on conformational dynamics. Inclusion of solvent accessibility for two surface hotspots, alongside one predicted APR, significantly improved models for predicting aggregation kinetics over 49 formulation conditions. The molecular-level insights gained will be important for guiding protein engineering strategies aimed at modulating these interactions to enhance product stability and retain therapeutic efficacy.

## Graphical abstract

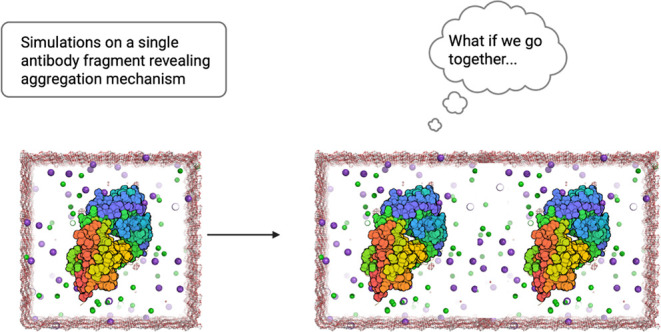

## Introduction

Protein–protein interactions (PPIs) play a pivotal role in numerous biological functions, including signal transduction, immune responses, and enzymatic regulation.^1^ The identification of specific regions, known as “hotspots,” that govern protein–protein interactions (PPIs) is crucial for elucidating the underlying mechanisms of these interactions, for developing strategies to modulate them, as well as for guiding protein engineering.^1–3^ Hotspots are typically defined as amino acid residues that contribute significantly to the binding free energy of PPIs. Identifying these residues is vital for understanding binding mechanisms and designing therapeutic interventions.^3,4^

Traditional experimental methods, such as alanine scanning mutagenesis, have been widely employed to identify hotspots. However, such approaches are often labor-intensive, low-throughput, and not always feasible for large or complex systems.^4^ Consequently, computational approaches have been developed to predict hotspots, enhancing the efficiency and scope of PPI studies.^5^ Computational tools, such as HotPoint, utilize solvent accessibility and pair potentials to predict hotspots in protein interfaces with reasonable accuracy.^1^ Similarly, machine learning algorithms have been applied to infer PPI hotspots, leveraging features like sequence and structural information to improve prediction performance.^6^ These *in silico* methods offer a high-throughput alternative to experimental techniques, enabling the analysis of large data sets and the generation of hypotheses for further experimental validation.

Molecular dynamics (MD) simulations have emerged as a powerful tool to investigate these interactions at an atomic level, providing insights into the frequency, duration, and randomness of contacts between protein interfaces.^7^ MD simulations complement predictive tools by providing dynamic information on PPIs. Studies have employed all-atom MD simulations to explore the dynamics of protein–protein complexes, revealing how conformational changes influence interaction stability and identifying transient versus stable contacts.^8–10^ For example, Kralj et al.^11^ employed MD simulations to investigate the interactions between an IgG1 antibody and various Fc gamma receptors (FcγRs), key players in immune responses. The simulations revealed novel interactions between the antibody Fab region and FcγRs, challenging the conventional understanding that binding occurs solely through the Fc region. Martin et al.^12^ used MD simulations to investigate the dynamic nature of protein–protein interactions in eight different protein complexes, revealing that their interfaces are not static but instead explore several stable, long-lived structural arrangements, even in complexes considered to be rigid bodies. These rearrangements, affecting both core and peripheral interface residues, involve changes in direct residue contacts and the behavior of interfacial water molecules.

Hotspots identified as important in PPIs have the potential to be used in predictions of aggregation, opalescence, precipitation, or solubility. Notably, surface-based aggregation prediction models have emerged, such as the spatial aggregation propensity (SAP) method.^13,14^ SAP used all-atom molecular dynamics simulations on a full antibody for 30 ns to identify regions based on exposed hydrophobic surface patches, helping to evaluate developability risks during antibody formulation.^13,14^ Others have explored the role of surface-exposed regions in aggregation mechanisms, particularly in the context of stress and formulation conditions.^15^ These approaches underline the growing importance of surface exposure and residue-level environment in understanding protein stability and aggregation behavior.

Complementary to these surface-based strategies, proteins are known to contain many buried aggregation-prone regions (APRs) with the potential to form cross-beta sheets^2,8,16^ that can become solvent-exposed in near-native protein conformations. Indeed, extensive biophysical and computational studies for Fab A33 have shown that these types of events influence aggregation kinetics.^17–23^ Fab A33 contains 214 residues on the light chain and 228 residues on the heavy chain. The stability, aggregation kinetics, and structural changes for Fab A33 have been studied extensively across many formulation conditions and through mutagenesis.^17^ Mechanistic modeling of experimental kinetics from Fab single mutants has previously shown that Fab A33 aggregation kinetics were rate-limited by a partial unfolding event to a native-like state.^19^ Such partial unfolding has the potential to expose one or more aggregation-prone regions (APRs),^17,24^ such as the seven predicted in Fab A33 as the consensus from four sequence-based algorithms: PASTA 2.0,^2^ TANGO,^16^ AGGRESCAN,^8,9^ and MetAmyl.^10^

The importance of partial unfolding events that expose these normally buried APRs, triggering self-association under stress conditions, was recently demonstrated by predictive modeling of the Fab A33 aggregation kinetics.^24^ This work established a predictive model of aggregation kinetics under 49 formulation conditions using both molecular dynamics simulations and machine learning, leveraging the contributions of APR exposure to predict experimental aggregation rates with promising accuracy. However, that study did not evaluate the potential contribution of PPIs formed through surface residue contacts within the native-like population.

The present study builds upon both the surface-based hotspot models and our previous APR-based framework to develop a more comprehensive model of antibody aggregation. It also aimed to further understand the molecular-level mechanism of the protein–protein interaction and offer a proof-of-principle study, demonstrating the power of MD simulations in investigating this phenomenon. Specifically, we aimed to explore: (i) the location of key interaction hotspots, (ii) the frequency and randomness of contacts, and (iii) the duration and stability of interaction events. By identifying consistent and high-frequency contact regions and comparing the behavior to single Fab systems, we aimed to uncover dynamic molecular patterns that influence aggregation kinetics in a range of formulations. These insights can inform rational antibody engineering strategies and support the design of robust therapeutic protein formulations.

We used all-atom molecular dynamics simulations to investigate the molecular mechanisms of protein–protein interactions for the A33 Fab antibody fragment (Fab A33). Given the constraints of current high-performance computing capabilities and the fixed time frame of this study, we limited the simulations to two Fab molecules but initiated them from 16 different randomized positions to increase the potential sampling of PPIs while reducing any bias due to the starting positions. As a key aim was to identify potential PPIs to include in predictive models for the aggregation kinetics determined previously across 49 conditions, the two-Fab system was simulated under two formulation conditions that spanned the experimental space.

Various methods were used to analyze the simulation data, including angle and distance analysis, principal component analysis (PCA), frequency contact map analysis, feature calculation, and SHapley Additive exPlanations (SHAP) analysis. Finally, the previous model,^24^ which predicted the aggregation propensity of Fab A33 from the solvent exposure of predicted APRs within single Fab MD simulations, was refined with the insights learned from the new protein–protein interaction investigation to achieve increased prediction performance using fewer input features (Scheme 1).

**Scheme 1:**
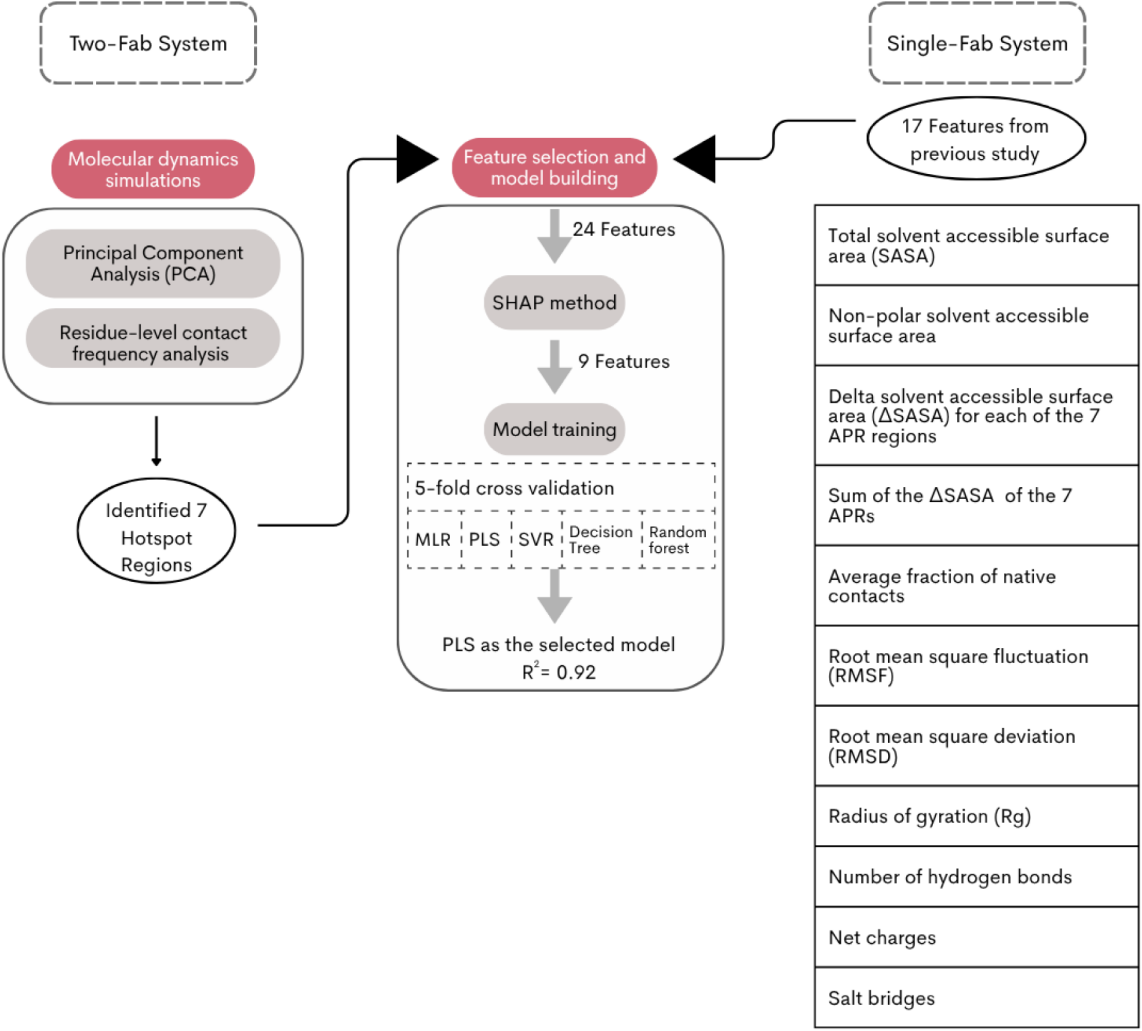
An Overall Workflow Including Molecular Dynamics Simulations, Principal Component Analysis (PCA), Residue-level Contact Frequency Analysis, Feature Selection, and Predictive Model Building Employed in This Work

## Materials and Methods

### All-Atom Molecular Dynamics Simulations

Molecular dynamics (MD) simulations were performed using Gromacs MD software, version 2019.3 ^25,26^ as previously described^24^ but on a system that contained two identical antibody fragments (2.5 Å resolution Fab A33 crystal structure (PDB ID: 7NFA), with 16 different starting positions at 338 K (65 °C) for 100 ns, in two different formulations: (i) pH 3.5, 0 mM NaCl; (ii) pH 7, 50 mM NaCl. The starting positions of the two antibody fragments were randomized manually before the simulation setup. A cubic box was used for defining the boundaries of the systems, and all of the proteins were separated by 10.0 Å from the box boundaries. The volume of the box was 3174.43 nm^3^ and the concentration of the protein was 6 mg/mL. Energy minimization of each system was performed using the steepest descent method to achieve a maximum force of less than 1000 kJ/mol/nm, followed by equilibration for 100 ps in the constant-volume ensemble (NVT) to stabilize at the specified temperature, and 100 ps in the constant-pressure ensemble (NPT) to stabilize at atmospheric pressure. The OPLS force field and extended simple point charge (SPC/E) water model were used to ensure consistency with previous work on Fab A33 and to future-proof studies with more complex systems. All simulations were run on the UCL Kathleen cluster.

### Angle and Distance Analysis

Vectors and central residues were defined in each Fab and used to calculate the relative angles and distances between the two Fabs. Both vectors (in Fab A and Fab B) were defined using the C atom of residue Arg30 and the C atom of residue Asp210. Residue Valine (residue index 163) in each Fab was selected as the representative to calculate the distance between the two Fabs throughout all the frames (Figure 1). All frames from 16 different starting positions of the MD simulations were combined, and a distribution of the distance of all the frames was generated and binned into 25 groups. The angle between the two defined vectors was calculated throughout all of the frames, and a distribution function of the angle was plotted for each bin group of the 25 distance ranges.

**Figure 1:**
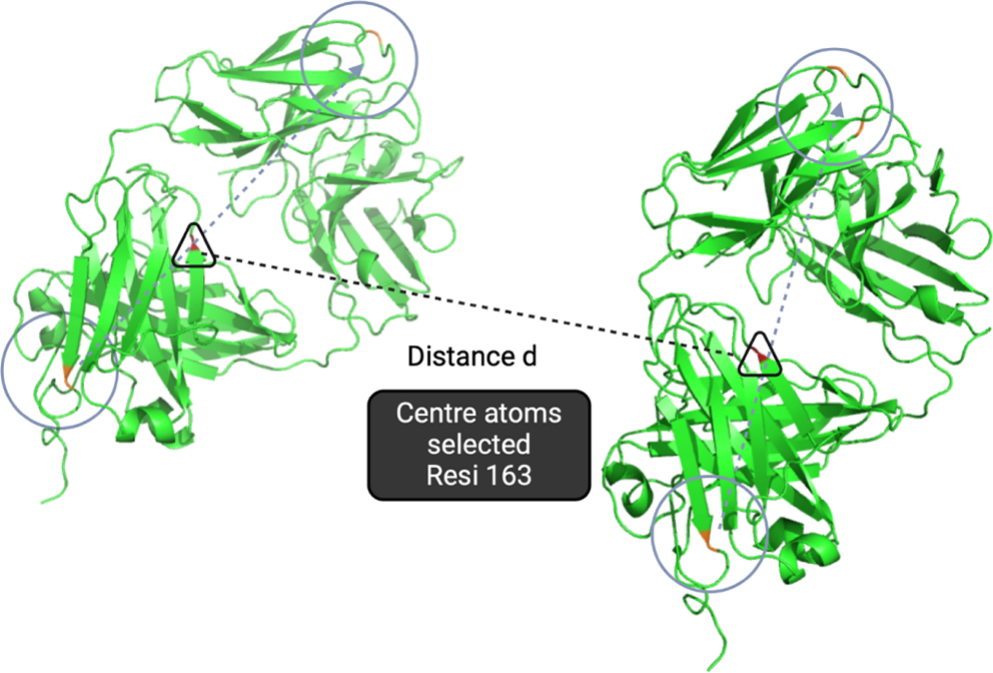
Visualization of the distance and angle calculation between two Fab fragments (PDB ID: 7NFA). These geometric parameters are used to characterize the relative orientation and spatial separation of the two Fab fragments, which may influence molecular interactions or structural stability. Vectors used for angle measurements are represented as dashed arrows, defined by the residues in the circled areas, and the central atoms (residue 163) selected for distance calculations are marked with triangles.

### Principal Component Analysis (PCA)

A principal component analysis (PCA) was conducted on all frames for which the two Fab molecules in the system made direct contact (threshold: <6 Å) from all the trajectories originating from different starting positions. Frames were clustered into different groups based on their contact locations. The PCA was performed using the Bio3D package.^27^ The covariance matrix was calculated from the data set containing all the atom coordinates from the trajectories, and eigenvalues and eigenvectors from the covariance matrix were obtained. The number of clusters was determined by the ability to cover the percentage of variance (selection threshold: covering 97% of the variance). A total of 9 clusters were chosen, and PCA was performed accordingly. The midpoint frames of the 9 clusters were extracted, and the interaction locations of the two Fabs were recorded.

### Frequency Contact Map Analysis

A frequency contact map analysis was conducted to record the residues in contact between the two Fabs during the simulations. For each frame in the simulations, the distance between each single residue in one Fab and all of the residues in the other was calculated, and a threshold of 6 Å was used to determine whether there was any contact between residues. A 442 × 442 matrix was established. If the distance between the two residues was smaller than 6 Å, a contact was recorded. For each single residue on the first Fab, if there was at least one contact with any other residue on the other Fab, it was counted as 1 for the residue. This process was repeated for each frame, and the residue counts were summed across all of the frames. A higher score indicates a higher contact frequency for the residue. The contact frequencies were calculated for each Fab separately and then combined.

### Feature Calculation

A total of six different features were calculated for all the MD simulations, namely root-mean-square deviation (RMSD), radius of gyration (*R*_g_), root-mean-square fluctuation (RMSF), total solvent-accessible surface area (SASA), nonpolar SASA, and the number of hydrogen bonds (defined in Table 1). Additionally, 7 regions that have the most frequent contact between the two Fabs, based on the frequency contact map analysis, were labeled as hotspot regions, and the time-averaged SASA of those areas was calculated. The SASA of the 7 APR regions was also calculated. The same features were calculated for a single-Fab system under the same conditions with six replicates to compare the features between the two-Fab and single-Fab simulations and to understand the impact of the other protein on the Fab. All features were calculated from the last 80 ns of the simulations.

**Table 1:** Molecular Features, Their Categories, Descriptions, and the Software Tools in Which They Were Calculated

Number	Name	Molecular descriptor code in the models	Category	Description	Software to calculate
1	Total solvent accessible surface area (SASA)	Total SASA	Geometrical	The total accessible areas of the solvent molecule on the surface of the α carbon atoms of the protein	GROMACS
2	Nonpolar solvent accessible surface area	Nonpolar SASA	Geometrical	The accessible areas of the solvent molecule on the surface of the nitrogen (N) and oxygen atom (O)	GROMACS
3–9	Delta solvent accessible surface area (ΔSASA) for each of the 7 APR regions	r_31–36 r_47-51r_114–118r_129–139r_261–265r_325–329r_387–402^a^	Geometrical	The ΔSASA within each APR was obtained by calculating the values averaged over the last 80 ns and subtracting the values averaged from the first 20 frames.	GROMACS
10–16	Delta solvent accessible surface area (ΔSASA) for each of the 7 hotspot	r_26–28 r_126-128r_152–153r_288–290r_348–352r_405–411r_437–442^b^	Geometrical	The ΔSASA within each hotspot was obtained by calculating the values averaged over the last 80 ns and subtracting the values averaged from the first 20 frames.	GROMACS
17	Root mean square fluctuation (RMSF)	Mean RMSF (last 80 ns)	Spatial (dynamic properties)	It measures the average deviation of a protein residue over time from a reference position	GROMACS
18	Root mean square deviation (RMSD)	Last 80 ns mean Rg	Spatial (dynamic properties)	It measures how much a certain molecular structure deviates from a reference structure	GROMACS
19	Radius of gyration (Rg)	Last 80 ns mean RMSD	Spatial (dynamic properties)	The radius of gyration measures the compactness of a protein structure.	GROMACS
20	Number of hydrogen bonds	Number of hydrogen bonds in the last 80 ns	Topological	The number of hydrogen bonds over time	GROMACS
21	Net charges	Net charges	Electrostatic	The net charge of the protein at different pHs	PropKa
22	Salt bridges	Salt bridge average	Topological	The average of the salt bridge occurrence in the last 50 ns	MDanalysis
23	Sum of the ΔSASA of the 7 APRs	sum_aprsasa	Geometrical	The sum of the delta solvent accessible surface area values for the 7 APR regions	GROMACS
24	Average fraction of native contacts	Average native contact (last 80 ns)	Topological	The fraction of native contacts over time	MDanalysis

a r_x-y: residue numbers, indicating APR 1–7.

b r_x-y: residue numbers, indicating hotspots 1–7.

### Hotspot Feature Integration into Fab A33 Predictive Models across 49 Formulations—Pearson Correlation and SHAP Analysis

A list of 17 molecular features was previously calculated^24^ and used to build predictive models for experimentally determined aggregation kinetics in 49 formulation conditions for the same Fab A33. The average SASA of the seven newly identified hotspot regions of the single Fab system in 49 formulation environments was now also calculated, giving a total of 24 molecular features (Table 1). Pearson correlation analysis was used to investigate the linear/monotonic correlation between each single molecular feature calculated from MD and the experimentally measured aggregation kinetics ln­(*v*), and melting temperatures (*T*_m_).^28^ The SHapley Additive exPlanations (SHAP) method^29^ was used to rank-order the 24 molecular features.^30^ SHAP uses classic Shapley values to rank the importance of the input features and signpost the redundancy between different features. The 24 molecular features were used as input variables to refine the previous model^24^ and the SHAP value for each feature was calculated with the SHAP TreeExplainer.^31^ A k-fold cross-validation was used to evaluate the performance of different models and select the best model among them. Generally, for small data sets, the cross-validation approach using the entire data set is more robust than retaining a single hold-out data set for validation purposes.^32^ Model building was performed with the scikit-learn package.^33^

## Results and Discussion

Molecular dynamics (MD) simulations were performed at 338 K (65 °C), using systems containing two identical antibody fragments (Fabs) placed in 16 different randomly assigned initial positions within a 14.7 nm-sided solvated cube. The simulations were carried out under two formulation conditions to span the previous 49 experimental conditions. The first, pH 3.5, 0 mM ionic strength, was chosen to represent the observed high aggregation kinetics, while the second, pH 7.0, 50 mM ionic strength, represented neutral pH conditions with lower-than-average aggregation kinetics.^28^ Each simulation was run for 100 ns. Upon visualization of the simulations, it was observed that the Fabs explored different spaces within the simulation box and gradually approached one another. Interestingly, they did not immediately form stable contacts at the nearest regions or first point of contact. Instead, they rotated around each other, making many transient contacts until a preferred interaction interface was identified. At this point, they typically engaged and remained stably bound for the remainder of the simulation (Movie S1, Supplementary File). Our analysis below sought to identify the predominant surface interaction hotspots at both of the simulation conditions. A principal component analysis (PCA) was performed for both conditions, giving similar results, while a more quantitative residue-level contact frequency analysis was taken forward into predictive model building. However, we focused a deeper analysis of the trajectories mainly on the pH 3.5 condition, as this was the most prone to aggregation experimentally.

### Distribution of Relative Angles and Distances between the Two Fabs during Simulations

To investigate the relative orientation and angle of the two Fabs during the simulations, the distance between the central residues (residue 163 in each Fab) and the angle formed between the two defined vectors (vector 1 in Fab A; vector 2 in Fab B) were analyzed across 22,884 frames from all simulations at pH 3.5, 0 mM ionic strength. The distribution of inter-Fab distances revealed that the majority of frames were concentrated within the 80–160 Å range, with a notable decrease in frame counts observed beyond 160 Å. This is because the dimensions of the simulation box (147 Å per side) impose a physical limit on how far two Fab domains can separate, with the maximum distance—along the box’s space diagonal—being 212  Å (Figure S1, Supplementary File).

Further analysis of the angle distributions within each distance bin showed that at shorter distances (below 60 Å), the two Fabs tended to adopt more defined orientations, with narrower angular distributions, indicating preferred binding geometries. In contrast, at larger distances (60–120 Å), angular distributions became broader and more variable, consistent with weaker inter-Fab interactions or a lack of stable contacts (Figure S1). At distances above 120 Å, the angular distributions began to reemerge into more defined regions, though not as well-defined as those below 60 Å. This was due to the proximity of the protein across the periodic boundary. The distributions were less well-defined at the larger distances, most likely due to the direction to the nearest protein across the cube, whereby the distance across the box varies from 147 to 212 Å, as well as the potential for interaction with one protein within the same box and another across the periodic boundary.

### Principal Component Analysis (PCA) to Identify Interaction Hotspots

Principal component analysis (PCA) was used to classify the interactions between the two Fabs into distinctive groups based on their atomic coordinates. All frames (a total of 7426 for each condition) for which the closest approach between the two Fabs was <6 Å (any atom–atom distance) were preselected and then clustered by PCA. This identified nine distinctive clusters in both cases based on their interaction locations and the relative positions of the two Fabs. Among the 7426 frames at pH 3, 0 mM NaCl, 23% were grouped in cluster 1, making it the largest cluster of the nine, followed by cluster 6 (17.7%) and cluster 5 (14.9%) (Supplementary File, Figures S2 and S3). From the 7426 frames at pH 7.0, 50 mM NaCl, 36.4% were grouped in cluster 1, making it, too, the largest cluster of the nine, followed by cluster 4 (16.7%) and cluster 6 (13.4%) (Supplementary File, Figure S3B).

The midpoint frames of the clusters were extracted, and the residue-level interaction locations of the two Fabs were recorded. The Fab positions from the midpoint frames of each cluster are shown in Figure 2 at pH 3.5, and 0 mM NaCl, and were similar for the pH 7 simulation clusters. The residues involved in interactions between the two Fabs are annotated and also highlighted in red. The nine clusters included tail-to-tail contacts (Clusters 1, 2), head-to-head contacts (Clusters 3, 9), side-to-tail contacts (Clusters 5, 6, 7), side-to-head contacts (Cluster 8), and head-to-tail contacts (Cluster 4). As Clusters 1, 6, and 5 were the most populated, most contacts involved tail-to-tail and side-to-tail interactions between the two Fabs. This was an interesting observation and consistent with previous MD simulations and mutational analysis that have implicated a strong influence of the instability in the hinge (tail) region on the aggregation kinetics of Fab A33.^19^ However, the current contact analysis alone does not confirm that the interactions in the tail region are specifically those that lead to more rapid aggregate formation.

**Figure 2:**
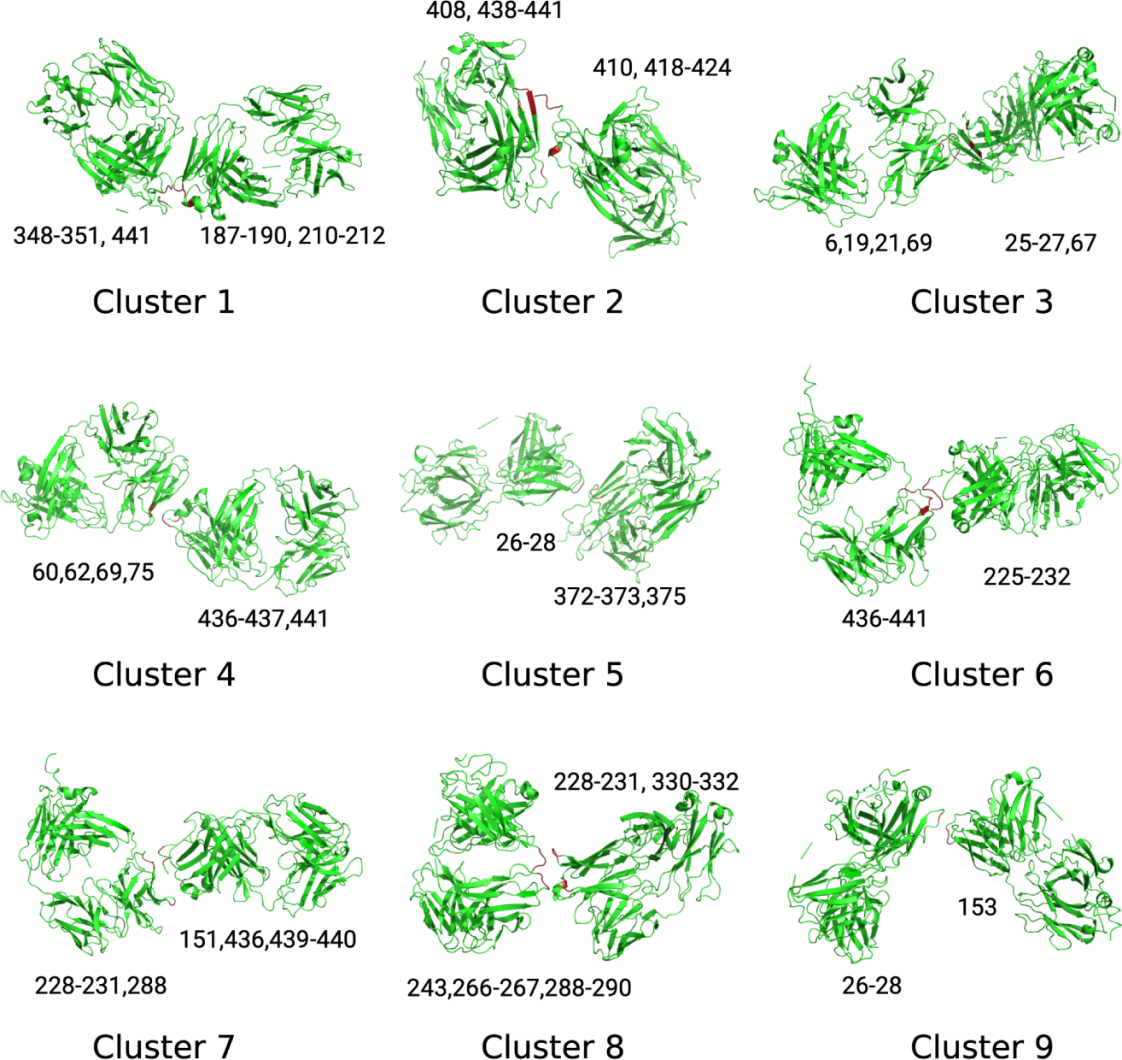
Hotspot regions identified by PCA are visualized in representative frames from the two-Fab simulations at pH 3.5. Contact regions are highlighted in red, with residue indices labeled.

In the analysis of the nine clusters, we noticed that several hotspot regions selected by the PCA were spatially adjacent within the 3D structure of the Fab. This proximity suggested that they may, in fact, represent a larger continuous interaction hotspot and so were combined into Hotspot Groups 1–5. Hotspot clusters located within 8 Å of each other were grouped into the same group (HG1–HG5), based on the proximity of their center-of-mass positions and guided by typical side-chain interaction distances observed in protein–protein interfaces. The relationships between PCA clusters, the specific residues involved, and the hotspot groups, as well as their relative occurrences, are depicted in Figure 3. For example, residues 348–352 from cluster 1 accounted for 23% of all observed contacts, while residues 436–441 from clusters 2, 4, 6, and 7 accounted for 24%. Given their spatial adjacency and shared residue neighborhood in the tail region, it is likely that these two regions act together to form a unified interaction interface, which we refer to as Hotspot Group 1 (HG1). When considered jointly, the combined hotspot is involved in approximately 47% of the total protein–protein interaction population—highlighting the overall importance of this tail region in Fab–Fab interactions. Similarly, residues 227–231 from clusters 6, 7, and 8 and residues 330–332 from cluster 8 were also found to be spatially adjacent, together accounting for 14% of the observed contacts. Their unified interaction interface was designated as HG2. HG2 was found to interact with residues 288–290 and 266–267 together (cluster 8), as well as with the HG1 region via clusters 6 and 7. Additionally, residues 410–411 from cluster 2 were spatially close to residues 372–375 from cluster 5. This interaction region was named HG3 and accounted for 11% of the observed contacts. However, residues 410–411 self-interacted with the other Fab, whereas residues 372–375 interacted with HG4 described next. Residues 26–28 formed HG4 alone and displayed significant contact activity across clusters 3, 5, and 9, accounting for 24% of the total contacts. This region was found to interact with both itself and the HG3 region. Finally, residues 288–290 from clusters 7 and 8 formed HG5 alone, accounted for 3% of the total contacts, and were found to interact with the HG1 and HG2 regions.

**Figure 3:**
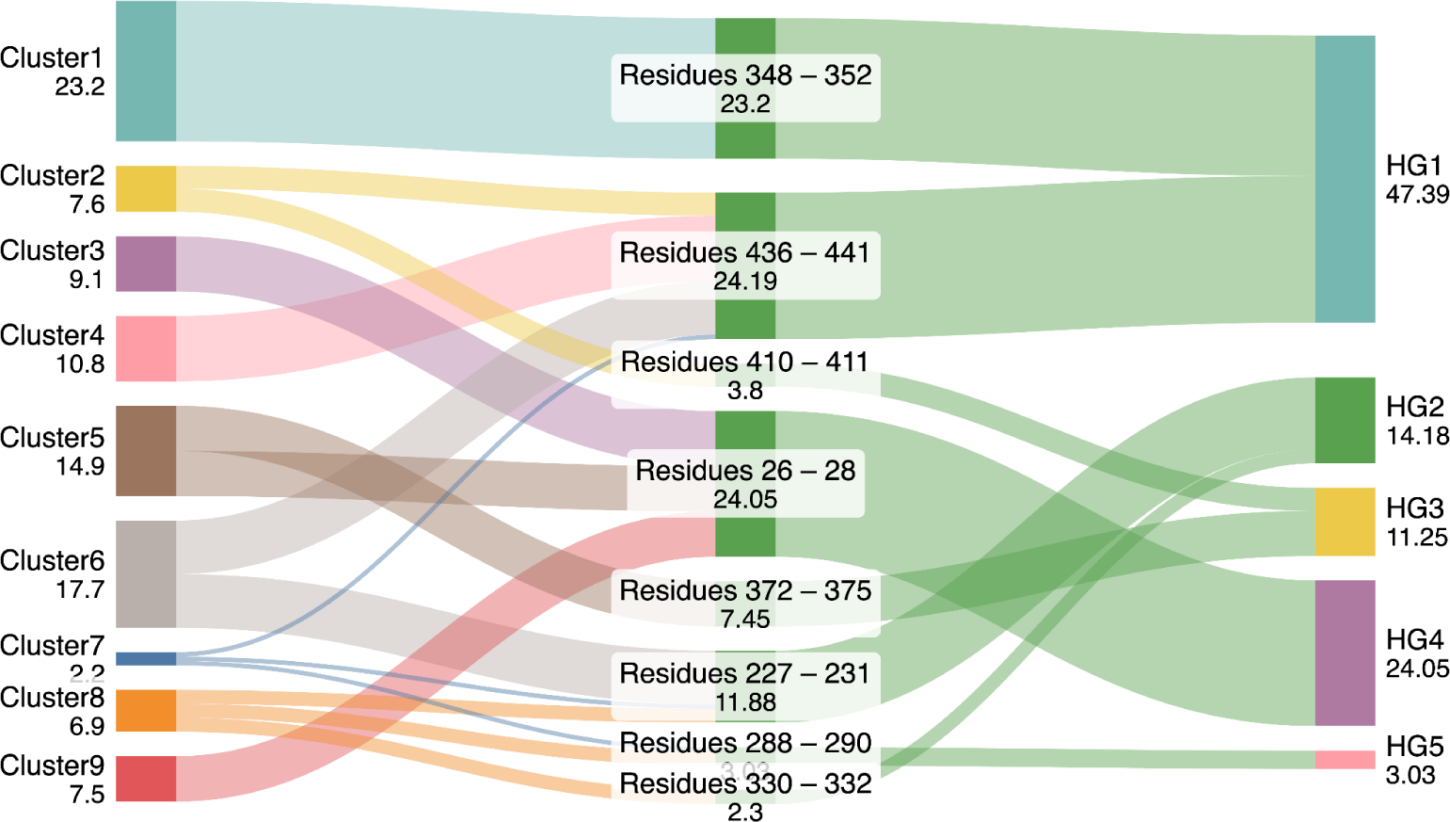
A flowchart illustrating the relationship between PCA-derived clusters, individual residue contact regions, and final hotspot groups (HG1–HG5) at pH 3.5 and 0 mM NaCl. Bands represent the flow of contact contributions from clusters to residue regions, and subsequently to unified hotspot groups. The band thickness corresponds to the numerical values and is (on a relative scale) indicative of the percentages shown for each hotspot group, which indicate their normalized contribution to the total contact interactions across all simulations. All numerical values shown in the figure, excluding the residue numbers, are percentages. Colors are used for visual clarity only and carry no further meaning.

Given that all interactions are a pairwise event between either two identical or two different Fab hotspots, it is important to note that the summed percentages of hotspot contact occurrences above were normalized to 100% to reflect their relative importance across all observed interactions. In other words, they are not simply the percentage of all frames in which a hotspot is involved in an interaction, as that would sum to greater than 100%. To account for overlapping contributions from multiple hotspot regions, the raw contact frequencies were normalized to reflect their relative importance across all observed interactions. This enabled a direct comparison of their relative interaction significance and reduced the potential bias introduced by shared residues or simultaneous contacts in two Fab simulations.

### Residue-Level Contact Frequency Analysis for Protein–Protein Interactions

To further validate the PCA-derived interaction hotspots and to obtain more quantitative data for predictive modeling, a residue-level contact frequency analysis was performed for both simulation conditions, in which residue–residue contacts between the two Fabs were recorded throughout the simulations using a 6 Å distance cutoff. The contact frequency accumulated over all the simulation frames was calculated for all 442 residues in each of the two Fabs, and the values were mapped by color intensity (white minimum to red maximum) onto the structure of the two Fabs, as shown in Figure 4 for the pH 3.5 condition. Several red regions in this figure overlapped with or closely neighbored one or more of the seven APR regions of the Fab A33 (black outline), highlighting the potential for links between the surface hotspot interaction sites and the exposure of buried APR regions.

**Figure 4:**
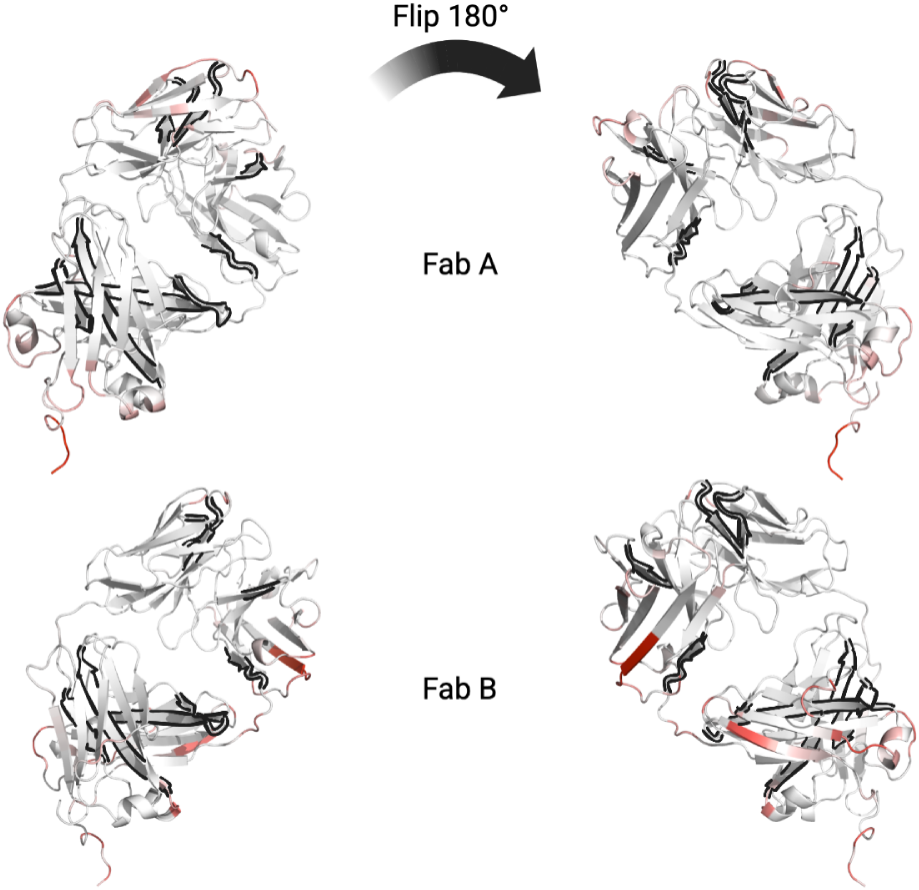
Structural distribution of residue-level contact frequency values for Fab A and Fab B at pH 3.5 and 0 mM NaCl. Contact frequencies are colored from the maximum frequency in red to the minimum frequency in white, with APR regions outlined in black.

Based on the contact frequency values of all 442 residues in each Fab, seven high-frequency regions were identified as hotspot regions at pH 3.5, and 0 mM NaCl (Supplementary File, Table S1). The selection criterion was that each region must consist of at least two residues with high contact occurrences in both Fabs. The contact frequencies were then averaged together over the two Fabs (Figure 5) to provide the final quantitative measure of how frequently specific residues were involved in contacts across all frames at each simulation condition. This confirmed the seven hotspots at pH 3.5 to be residues 26–28 (V_L_), residues 126–128 (C_L_), residues 152–153 (C_L_), residues 288–290 (V_H_), residues 348–352 (C_H_), residues 405–411 (C_H_), and residues 437–442 (C_H_), averaging >750 contacts in total. These same hotspots were observed at pH 7, but one additional hotspot stood out at residues 187–190 (C_L_) with >1250 contacts in total. This hotspot was observed at pH 3.5 but with a much lower frequency of <500 contacts in total.

**Figure 5:**
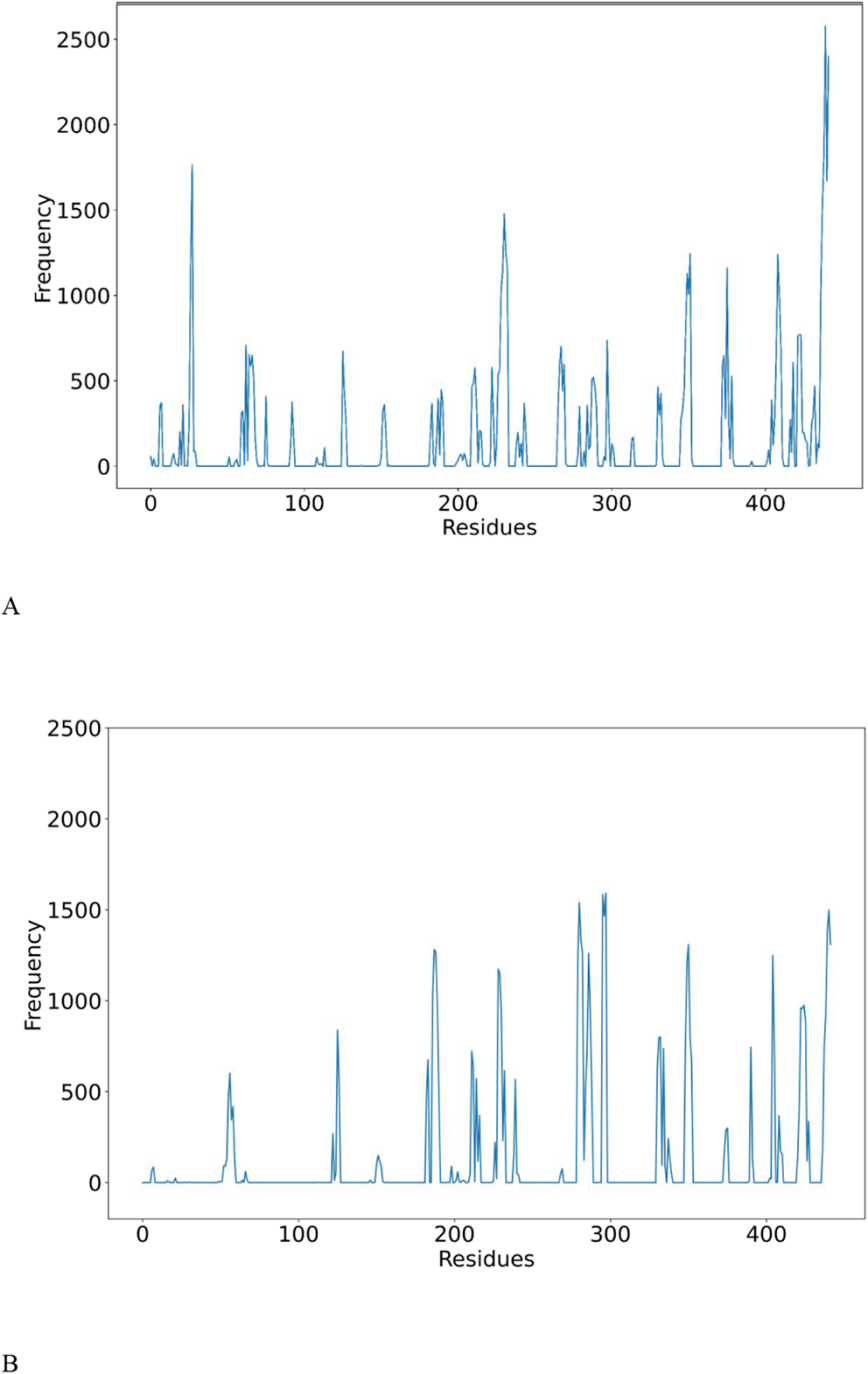
The total frequency of residue–residue contacts for all 442 residues in Fab A33, combined from both Fab A and Fab B across 16 different starting positions at pH 3.5, 0 mM NaCl, and 338 K (A), and pH 7, 50 mM NaCl, and 338 K (B). The residue–residue contacts between the two Fabs were recorded throughout the simulations by using a 6 Å distance cutoff.

Comparison with the hotspots identified from the PCA confirmed that the major peaks in the frequency contact map corresponded very well to the key interacting residues identified within the nine representative clusters from PCA. This agreement between residue-level contact frequencies and structural clustering suggests that the high-frequency contacts are not artifacts of transient interactions but instead represent stable, recurrent interfaces formed during the simulations. Furthermore, this consistency supports the validity of using principal component analysis (PCA) as a means of identifying dominant motions and capturing structurally significant contact patterns. The ability of PCA to highlight residue–residue contacts that occur at high frequency reinforces its utility in detecting relevant molecular interactions, particularly in systems where dynamic conformational sampling plays a central role.

A population distribution of all the residue-level contact frequencies over the simulations is shown in Figure S4. This shows that the majority of residues had contact frequencies of fewer than 150 frames (from 7426 contacting frames). Most, though not all, of this can be explained by residues that are buried away from the Fab surface and make zero contacts. However, approximately 100 residues had between 1 and 271 contacting frames, indicating surface residues for which any contact is very short-lived. By contrast, the higher frequency contact sites were relatively few and suggested more stable interaction hotspots rather than random transient occurrences. A further analysis of the duration of contact events (Figure S5) showed that the formation of clusters was very stable (i.e., continuous contacts at the same regions), with events lasting between 428 and 1316 frames (4.2–13.2 ns). Interestingly, on several occasions, the contact position shifted between two clusters in a transitional event without completely breaking contact. This happened between clusters 1 and 2, clusters 5, 8, and 9, and clusters 6 and 7, and is consistent with the spatial proximity of residues in contact within each of these cluster groups. Clusters 1 and 2 are linked via Hotspot Group 1 (HG1), clusters 5 and 9 are linked by residues 26–28 in HG4, and clusters 6 and 7 are linked via HG1 and HG2. This observation confirms the significance of the hotspot groups as neighboring regions of contact residues that act together such that the protein–protein interaction is dynamic, moving readily between slightly different contacts within the same overall region without losing contact altogether. Together, this also served to demonstrate that the interaction sites shown in Figures 2–4 generated from the principal component and contact frequency analyses, were unlikely to be random occurrences.

### Impact of Protein–Protein Interactions on Global Conformation and Dynamics

It was considered potentially informative to evaluate whether the formation of contacts at the surface hotspots could influence other properties of the protein, such as the global and local structure or dynamics. Six features, including RMSD, total nonpolar SASA, total SASA, *R*_g_, and the number of internal hydrogen bonds, were calculated from the 100 ns MD simulations initiated at 16 different starting positions for the two Fabs. The same features were calculated from the single-Fab simulations under the same conditions, each with six replicates as well, to compare the features between the two-Fab and the single-Fab simulations and to understand the impact of the second protein on the Fab. The changes in each feature over the simulations were further classified into three subgroups: frames in contact, frames with no contact, and frames from the single-Fab simulations only. The six features are shown in Figure S6 in the Supporting Information as whisker-box plots.

Among the six features, RMSD, *R*_g_, and the number of hydrogen bonds do not display significant differences among the frames in contact, the frames that had no contact, or the frames from the single-Fab simulations. The average RMSF did show some decreases when comparing the Fab in contact with the second Fab to the Fab from single-Fab simulations. These suggest that the formation of contacts results in lower overall flexibility or fluctuational dynamics within the Fab protein.

The total SASA was found to exhibit a more significant difference between frames in contact and those in the noncontact conditions or the single-Fab system. The contact frames consistently showed lower SASA values compared to the other two groups, with the upper quartile of the contact frames being lower than the lower quartiles of the other two groups. This pattern indicates that the contact condition led to a significant burial of the protein’s surface in the interaction interface, reducing the overall exposure of the protein to the solvent. This observation was consistently reflected in both Fabs, suggesting that the effect was not random. The nonpolar SASA also displayed certain differences between frames in contact and those in the noncontact conditions or the single-Fab system, with the contact frames showing slightly lower values, but the differences were not as significant as those in the total SASA. The lower total SASA values in the contact frames suggest that a significant proportion of the protein surface became less exposed to the solvent during protein–protein interactions. Combined with the observation that the nonpolar SASA values changed less significantly, it might indicate that the interactions were likely hydrophilic or polar interactions, and their surface residues were shielded from the solvent, resulting in a significant decrease in total SASA. The noncontact conditions and the single-Fab condition, exhibiting higher SASA, suggest a more flexible or extended conformation of the protein, with a larger surface area exposed to the solvent. This is consistent with the behavior of proteins in unbound or flexible states, where greater solvent accessibility is often observed.

### Impact of Protein–Protein Interactions on Predicted APR and Surface Hotspot Solvent Accessibility

Aggregation-prone regions (APRs) are traditionally thought to play an important role in protein aggregation by exposing the cross-β sheet, which is more prone to self-interaction. This typically requires a degree of partial protein unfolding to reveal the APRs to the solvent. However, the surface hotspot regions that are key to PPI in the simulations might also contribute to aggregation kinetics. Therefore, the SASA of the seven predicted APR regions and the seven top hotspot regions found in the frequency contact map analysis were calculated and further classified into three groups, the same as the features calculated above: frames in contact, frames with no contact, and frames from the single Fab system. The values for the seven APR and seven hotspot regions were displayed as whisker-box plots in Figures S7 and S8 in the Supplementary File.

Of the seven APRs, residues 129–139 (C_L_) displayed differences between frames in contact and those in the noncontact conditions or the single Fab system, with the SASA values for the frames in contact being lower than those in the other two groups. However, this difference was not significant enough to indicate that the APR region was buried during all the different types of interactions but was likely to be buried in certain interactions between the two Fabs. Similarly, from the seven hotspot regions, residues 348–352 (C_H_) and residues 405–411 (C_H_) also displayed lower SASA values for frames with two Fabs in contact.

The SASA for the seven hotspot regions was also calculated from frames within each of the nine clusters generated by PCA (Figure S9, Supplementary File). Clusters 3 and 7 displayed a lower SASA value in the hotspot region at residues 288–290 (V_H_) than the other clusters, indicating that the interactions within those two clusters buried the residues 288–290 (V_H_), resulting in low SASA values. Similarly, the hotspot regions at residues 348–352 (C_H_) and residues 405–411 (C_H_) were more buried in clusters 5, 6, and 8, while the hotspot region at residues 26–28 (V_L_) was more buried in cluster 1. Overall, these results indicate that protein interactions, as represented by each cluster, led to decreases in solvent accessibility in only one or two hotspots at most.

### Hotspot-Enhanced Model for Prediction of Aggregation Kinetics

The current simulations were carried out at two formulation conditions that spanned the range of conditions and aggregation kinetics observed previously over 49 conditions. The two conditions led to accelerated aggregation kinetics (pH 3.5, 0 mM NaCl, 338 K (65 °C)) and lower-than-average kinetics at a pH more representative of typical formulations (pH 7.0, 50 mM NaCl, 338 K). It was possible that the hotspots identified might have different degrees of importance and influence on the structure and dynamics under different formulation conditions. As such, their associated solvent accessibility was hypothesized to be an important additional feature that could be used in predictive models for aggregation kinetics.

The PCA and contact frequency analysis found that seven hotspot regions were common across both of the conditions simulated, suggesting that they are very likely to be common to all 49 of the conditions previously simulated and experimentally tested. All seven of the hotspots identified at pH 3.5 were observed at pH 7, but one additional hotspot was identified at pH 7. Therefore, although the seven common hotspots were securely identified through repeats at two very different conditions, it was also very likely that we had captured the most important hotspots.

Our previous study used a regression model, PLS, to train a data set that included 17 features calculated from the MD simulations at 49 formulation conditions and to predict the aggregation kinetics, achieving an *R* ^2^ of 0.84. That model did not include any surface hotspot features but focused primarily on inherent conformational dynamics and exposure of buried aggregation-prone regions (APRs) to drive aggregation. Others have used surface-based hotspot analyses, such as SAP, to predict aggregation propensities on the assumption that aggregation is driven by contacts between surface features of the protein.^13,14,34^ As both the buried APR exposure and surface hotspot interactions appear to have a role in aggregation, we aimed to generate new predictive models that incorporated both elements.

Since the seven surface hotspot regions identified under both conditions, and the additional one identified at pH 7 only, were key to protein–protein interactions in the simulations, it was hypothesized that they might also contribute to aggregation kinetics under other conditions. Therefore, the SASA of the surface hotspots was added to the 17 previous features, with the aim of refining the previous model and increasing its predictive ability. The SASA for these surface hotspots shows significant variation across the 49 conditions explored in the previous MD simulations (Figure S10, Supporting Information), indicating their potential to contribute to a predictive model. Pearson correlation analysis and SHAP analysis were used on the feature data sets to demonstrate the correlation between features and feature ranking orders, with specific interest in the hotspot regions identified in the previous analysis. Furthermore, with the newly identified top-ranking features by SHAP analysis, the previous model was refined and optimized.

### Pearson Correlation

The correlation heatmap matrix shown in Figure 6, between several experimental and calculated features, presented a range from strong negative (black) to strong positive (off-white) correlations. Hotspot regions at residues 288–290 (V_H_) displayed a surprisingly high Pearson correlation coefficient (*r* = 0.80) among all the hotspot and APR regions when correlated with the aggregation kinetics, ln­(*v*). Moreover, its coefficient value was the third highest among all of the features, after RMSF (*r* = 0.84) and RMSD (*r* = 0.81). Hotspot regions at residues 126–128 (C_L_) and residues 437–442 (V_H_) also showed relatively high Pearson correlation coefficients (*r* = −0.58, −0.75). It was interesting that these hotspots were not the ones that had the greatest changes in SASA within the two-Fab simulations at pH 3.5, 0 mM NaCl, and 338 K (65 °C). This emphasizes how the relative importance of hotspots is likely to be different across widely varying formulation conditions. Interestingly, the hotspot at residues 187–190 that was identified at pH 7 only did not correlate well with the aggregation kinetics (*r* = −0.48), which suggests that it may not contribute well to a model for predicting the kinetics over the full 49 conditions.

**Figure 6:**
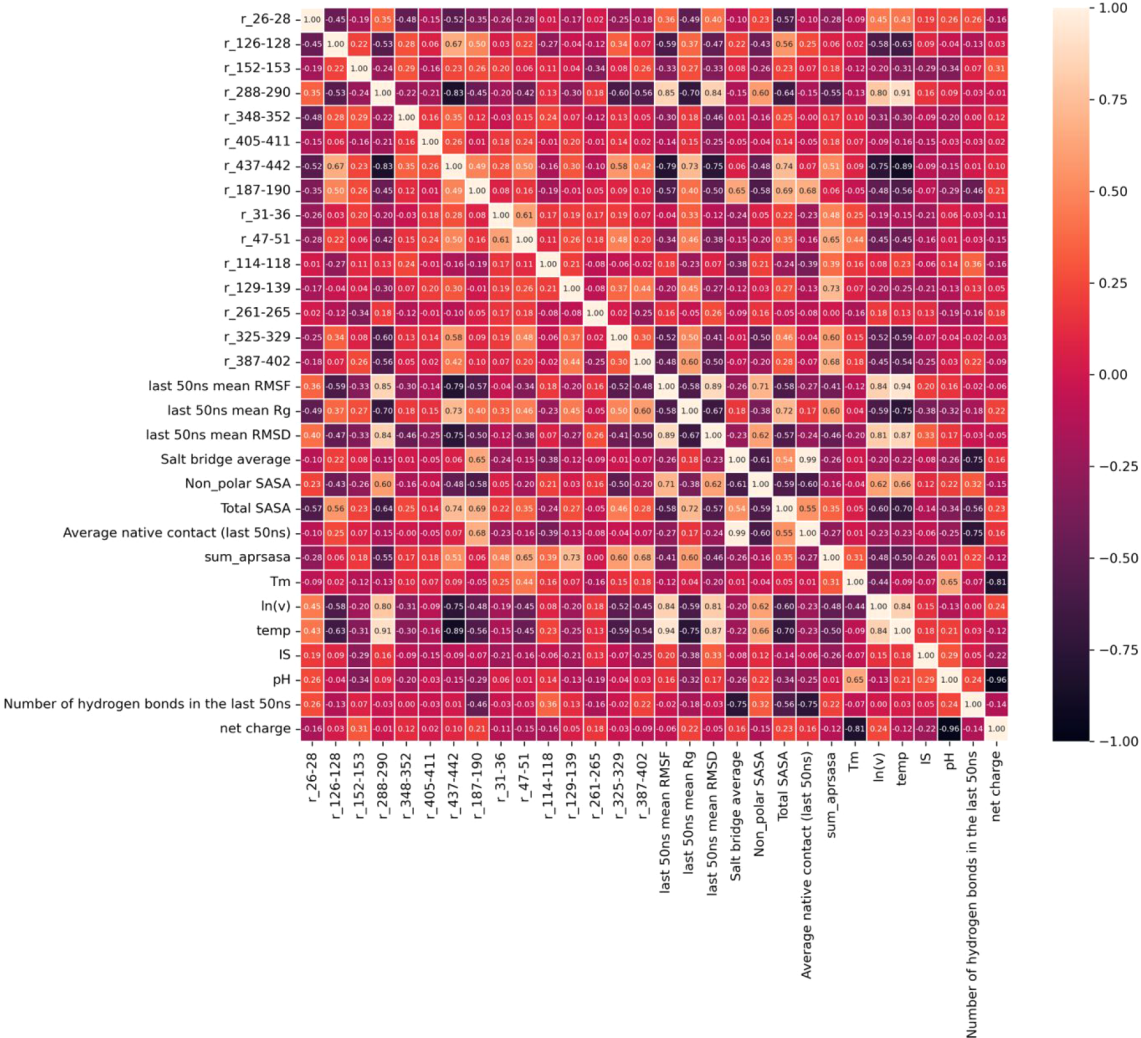
A heatmap of Pearson correlations (as *r* values) between all of the variable features, experimental variables (used as labels during model building), and experimental data of melting temperature (*T_m_*) and aggregation kinetics (ln­(*v*)) is shown. The color code on the right side indicates correlation strength, with black representing a strong negative correlation, while white or mild orange represents a strong positive correlation. A detailed description of the names of the variables can be found in Table 1. Hotspot region r_187–190 was unique to the condition pH 7, 50 mM NaCl, 338 K.

### SHAP Analysis

In previous studies,^25^ MD simulations under different formulation conditions were used to identify the APRs that might be relevant to aggregation kinetics. A SHAP analysis evaluated 17 features, including the solvent accessibility of the APRs, for their importance in predicting aggregation kinetics. However, the previous work did not include surface hotspot features, so we have now extended the analysis here to examine the potential role of hotspots alongside APRs and their contribution to predicting aggregation kinetics. The feature selection method was based on the SHAP TreeExplainer with the XGBRegressor model to rank high-contribution features. An initial SHAP analysis that included the unique hotspot at pH 7 (residues 187–190) ranked this feature only 10th (Figure S11, Supporting Information). Taking this into consideration, along with the low Pearson correlation, this feature was excluded, and the SHAP analysis was repeated (Figure 7). Four out of the top ten features were found to be hotspot regions, and two out of the top ten were APRs. The remaining four features in the top ten included a mix of both surface properties (SASA) and dynamic properties (RMSD, global average RMSF, *R*_g_).

**Figure 7:**
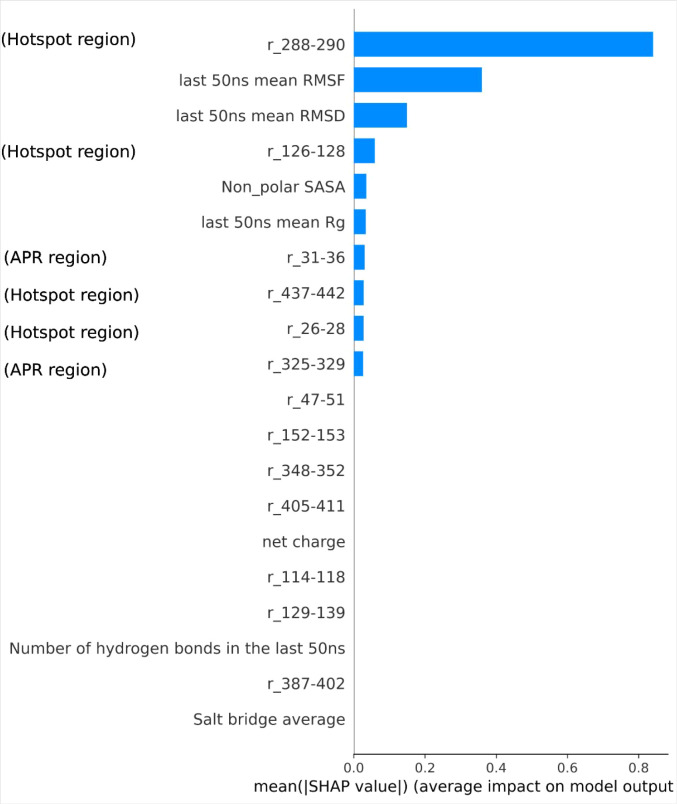
SHAP analysis showing the relative contributions of 24 molecular features to the XGBoost model. The bar chart represents the average impact of each feature on the model’s output, with longer bars indicating higher feature importance.

The new SHAP analysis showed the importance of the hotspot regions at residues 288–290 (V_H_), 126–128 (C_L_), 437–442 (V_H_), and 26–28 (V_L_). It also confirmed the importance of the APR region (residues 31–36, V_L_) in the previous finding. By contrast, some of the APR regions that were considered impactful (residues 129–139, C_L_; residues 114–118, C_L_; residues 261–265, V_H_) in the previous models became much less impactful when including hotspot regions. This could potentially reflect a degree of correlation between the structural dynamics of certain hotspots and that of nearby APRs, but where the surface hotspots have a more direct influence on aggregation kinetics than the nearby APRs. For example, the top-ranked hotspot region 288–290 is spatially close to APR region 261–265. The second top-ranked hotspot region 126–128 is sequentially close to APR region 129–139 and spatially close to APR region 114–118. Finally, hotspot region 26–28 is sequentially close to APR region 31–36, confirming their importance from models explored in both studies, and the hotspot region is also spatially close to APR region 47–51 (Figure 4). This finding offers promise for the future design of enhanced protein engineering.

### Model Building

The same regression models from the previous study were examined here, including multiple linear regression (MLR), partial least-squares (PLS), support vector regression (SVR), decision tree regression, and random forest regression from the scikit-learn package. A GridSearchCV with 5-fold cross-validation was used to select the best-performing model and optimize the hyperparameters. Since GridSearchCV uses cross-validation, the risk of overfitting is minimized. The PLS model includes one hyperparameter as the number of components. SVR has three hyperparameters, including C (regularization parameter; tested values were 0.1, 1, 10, 100), epsilon (tested values were 0.01, 0.1, 0.2), and kernel (“linear”, “poly”, and “rbf”). The optimal hyperparameters found through GridSearchCV were selected based on the *R* ^2^ score, and the best model was used for predictions. The top 9 features from SHAP analysis were selected, and a total of 511 possible subsets of these features were tested. The effect of different feature combinations on the model’s performance was examined. The best performance was achieved when using the SVR model with hyperparameters as “C”: 10, “epsilon”: 0.1, “kernel”: “rbf” and four input features, including two hotspots (r_288–290, r_126–128), last 50 ns mean RMSD, and the APR r_31–36, with an *R* ^2^ score of 0.92. This feature combination provided the best predictive power for the target variable, demonstrating a strong fit between the predicted and actual values of ln­(*v*). A parity plot was created to visualize the relationship between the actual values and predicted values of ln­(*v*) (Figure 8). The plot demonstrates that this model, using only four features, performs better (*R* ^2^ = 0.92) than the previous model^24^ which used nine feature combinations (*R* ^2^ = 0.84), with most predicted values closely matching the actual values. The red dashed line represents the ideal line where predicted values would equal the actual values, confirming that the model is successful in capturing the trend.

**Figure 8:**
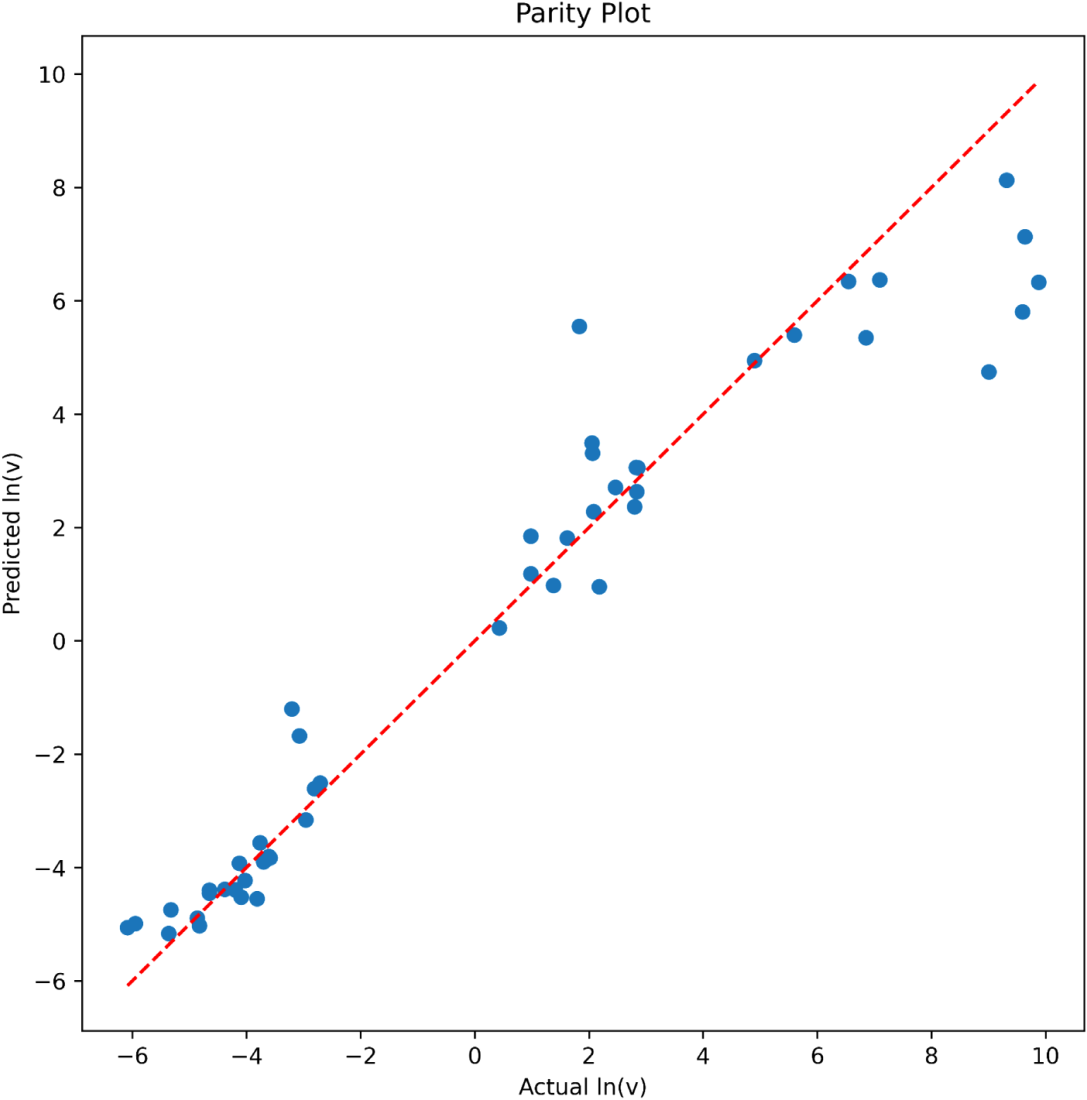
A parity plot showing the relationship between the actual and predicted values of ln­(*v*) using the best SVR model, with an *R* ^2^ of 0.92. The red dashed line represents the ideal fit, where predicted values match the actual values. This model used only four features: hotspot r_288–290, hotspot r_126–128, last 50 ns mean RMSD, and APR r_31–36.

### A Focused View of the Hotspot Regions at Residues 288–290 (V_H_)

To deepen our molecular understanding of Fab–Fab interactions, we focused on the hotspot region at residues 288–290 (V_H_), identified as the most important during model building. Example structures from the simulations, in which protein–protein interactions form via these hotspots, are shown in Figure 9. The first example from cluster 8 shows hotspots 288–290, with residues 267–268 and 243–245 in one Fab interacting with residues 184–190 and 211–212 in the other Fab. The second example, taken from cluster 7, shows that hotspot residues 287–290, along with residues 283, 298, and 299 from one Fab, interacted with residues 151–154 (including hotspot 152–153) and residues 441 and 442 (from hotspot 437–442) in the other Fab. In both cases, the total protein–protein interaction is significant, with 12 contacts made. Both cases also show that the interactions are not simply hydrophobic, as might be assumed. Indeed, the specific contacts were approximately evenly split, with the first example having 7 polar hydrogen-bonded and 5 nonpolar van der Waals interactions when thresholding at ≤5 Å, and the second example having 5 polar and 7 nonpolar interactions. The smaller-than-perhaps-expected role of hydrophobic residues in surface hotspot interactions is likely due to the relatively low number and inaccessibility of hydrophobic patches on the Fab A33 surface. An analysis using Protein–Sol^35^ revealed only one significant nonpolar patch formed from residues 223–225 and 324–328 in the heavy chain. However, these residues are within the elbow region and face inward to form a cleft in Fab that makes them essentially inaccessible for protein–protein interactions, and consequently were not identified as a hotspot. This low hydrophobicity most likely reflects the generally stable nature of Fab A33, whereby some of the 49 formulations have aggregation rates of <1% per year.

**Figure 9:**
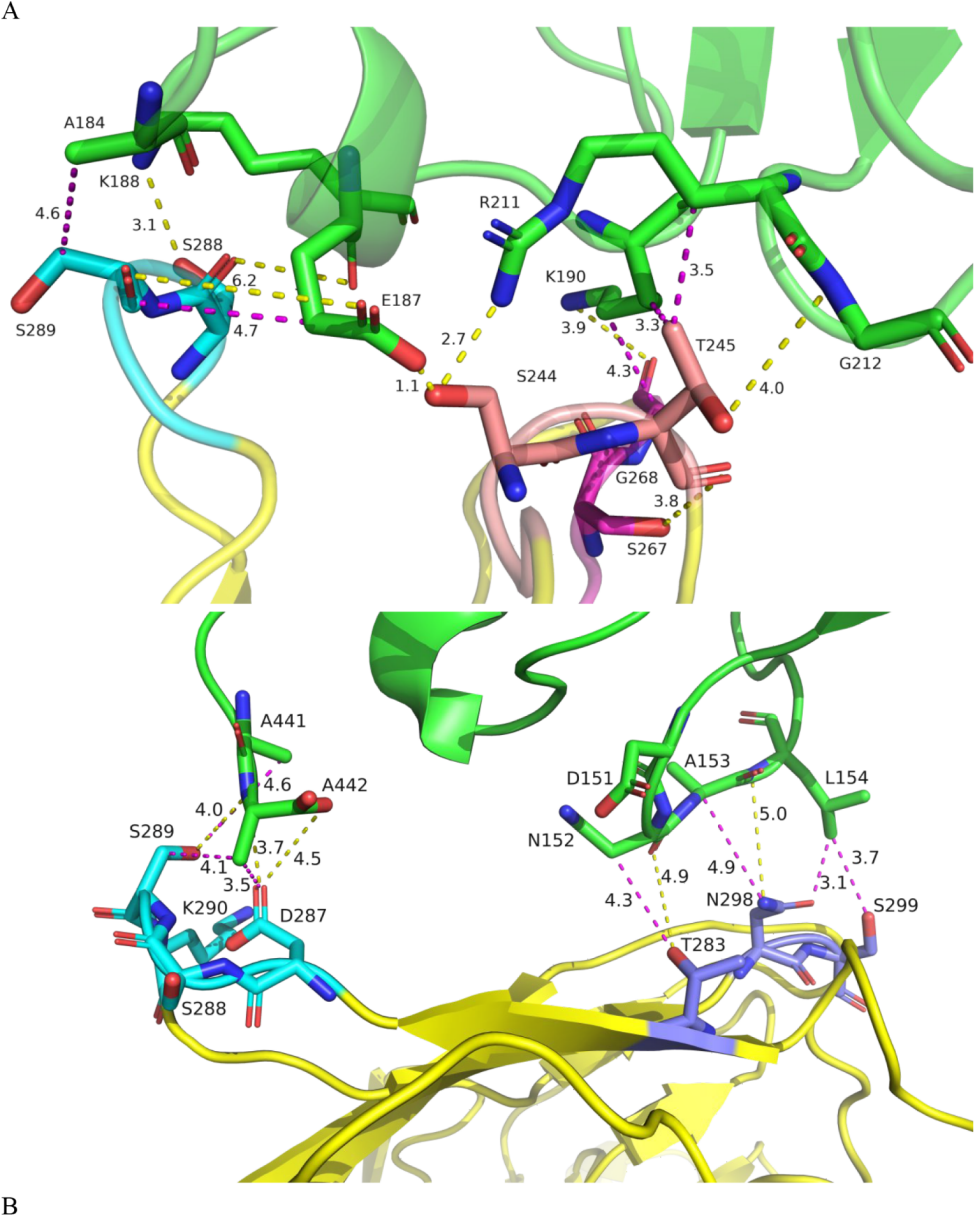
Typical interactions of hotspot regions. The two Fab molecules are shown as yellow and green cartoons. A) Hotspot 288–290 in cluster 8: interactions involve residues 288–290 (cyan), 267–268 (magenta), and 243–245 (salmon) from one Fab, with residues 184–190 and 211–212 (green) in the other Fab. B) Hotspot 288–290 in cluster 7: interactions involve residues 287–290 (cyan) and residues 283, 298, and 299 (blue) from one Fab, with residues 151–154 and residues 441 and 442 (green) in the other Fab. Polar hydrogen bonds are shown as yellow dotted lines, and nonpolar contacts are shown as magenta dotted lines.

## Conclusion

This study employed all-atom molecular dynamics simulations to investigate protein–protein interactions (PPIs) in two-Fab simulations, focusing on identifying key interaction hotspots and characterizing their dynamics. Through frequency contact map analysis and principal component analysis (PCA), we identified specific residues that form stable contacts, distinguishing them from transient interactions. These analyses revealed that certain regions act as persistent hotspots, influencing the overall interaction between the Fab fragments.

Further investigation through feature calculations, including solvent-accessible surface area (SASA) analysis of both aggregation-prone regions (APRs) and surface hotspots, provided insights into the structural changes during PPIs. Notably, the total SASA exhibited significant differences between contacting and noncontacting frames, indicating substantial surface burial during interactions. Furthermore, by incorporating hotspot features into a previously developed model, we refined its predictive ability for aggregation kinetics. This refined model, using only four key features, achieved an improved *R* ^2^ score of 0.92, demonstrating enhanced predictive power compared to the previous model.

These findings provide a molecular-level understanding of Fab–Fab interactions, emphasizing the role of specific hotspots in driving stable contacts and influencing aggregation. The insights gained can guide protein engineering strategies aimed at modulating these interactions to enhance therapeutic efficacy and formulation stability. Future studies could explore the impact of different environmental conditions on these interactions and further validate the identified hotspots through experimental approaches.

## Supplementary Material



## Notes

Dynamic visualization of the dual-Fab simulation (starting position 16, replicate 1) under formulation conditions: pH 3.5, 0 mM NaCl, 338 K (65 °C); angle and distance plot; Principal Component Analysis (PCA) of two Fab molecular dynamics simulations; population distribution of the nine clusters identified by principal component analysis (PCA); selected hotspot regions based on frequency contact occurrence; the distribution of contact frequencies between the two Fab fragments across all simulations and starting positions; occupancy of the nine PCA clusters over time; comparison of various molecular features between single Fab and two Fab simulations under contact and noncontact conditions; average solvent-accessible surface area (SASA) of seven hotspot regions across nine clusters at pH 3.5, and across all 49 formulation conditions; SHAP analysis showing the relative contributions of all the molecular features to the XGBoost model, including the extra hotspot region identified only at pH 7 (residues 187–190) (PDF)
