# Supplementary material for: Hotspot Interactions between Two Fab Molecules in Molecular Dynamics Simulations Improve Predictive Models of Aggregation Kinetics
Source: Mol Pharm. 2026 Feb 9;23(3):1722–36. doi: 10.1021/acs.molpharmaceut.5c01464 (PMC12958343; doi:10.1021/acs.molpharmaceut.5c01464)
Supplement: Supplementary file 1 [file mp5c01464_si_001.pdf]

Supplementary Information:

Hotspot interactions between two Fab molecules in molecular dynamics simulations improve predictive models of aggregation kinetics

Yuhan Wang[1], Hywel D. Williams[2], Duygu Dikicioglu[1], Paul A. Dalby[1]*

[1] Department of Biochemical Engineering, University College London, London, WC1E 6BT, UK

[2] CSL Ltd, Biopharmaceutical Product Development, 45 Poplar Road, Parkville, 3052 Australia

Movie S1. Dynamic visualisation of the dual-Fab simulation (starting position 16, replicate 1) under formulation conditions: pH 3.5, 0 mM NaCl, 338 K (65 °C).

https://drive.google.com/file/d/1WEdzdoxQ9ffL9pggHvpubfwvB_pojBKx/view?usp=sharing

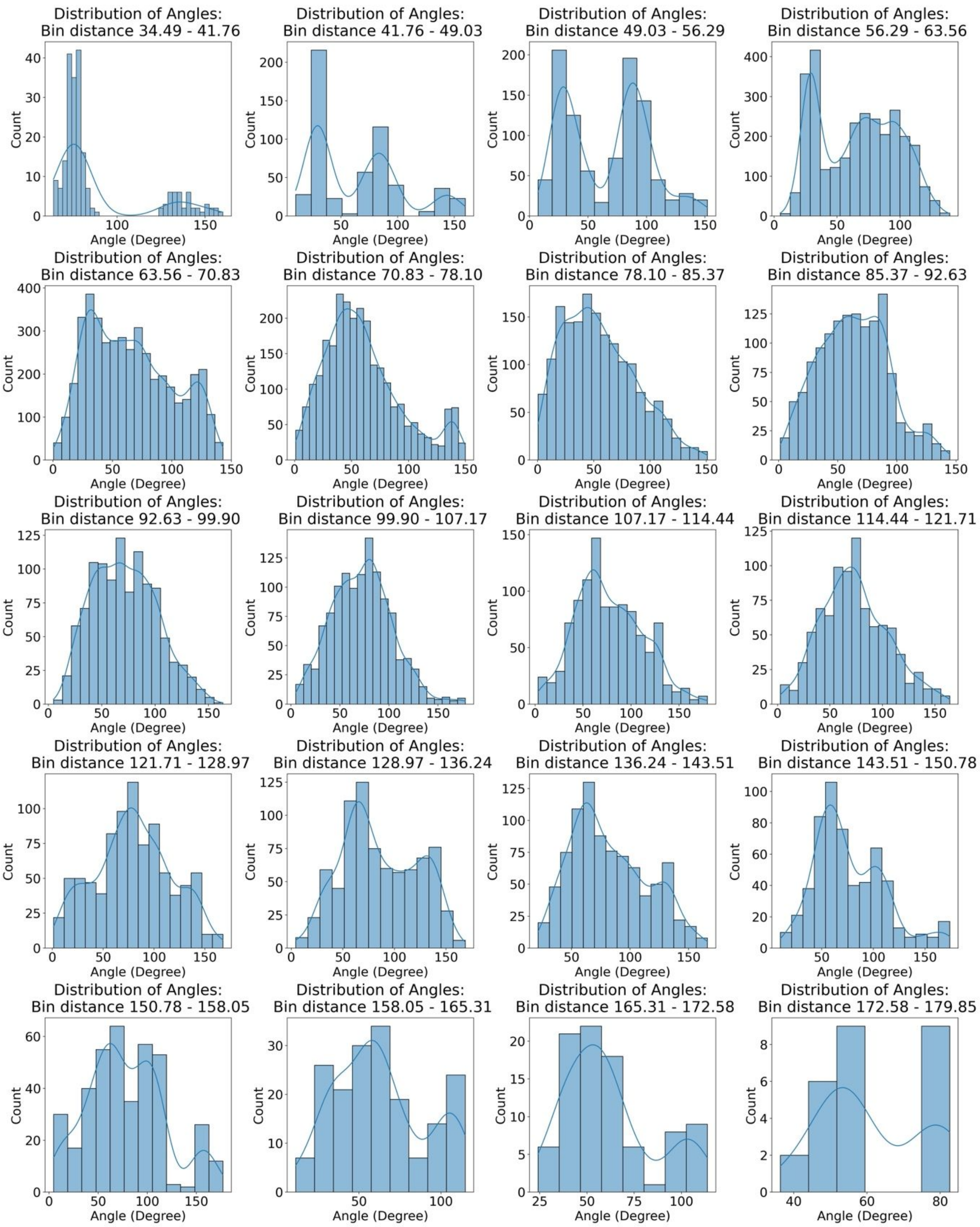

Figure S1. Angle and distance plot

Data from 22,884 frames from all simulations at 338 K, pH 3.5, 0 mM NaCl.

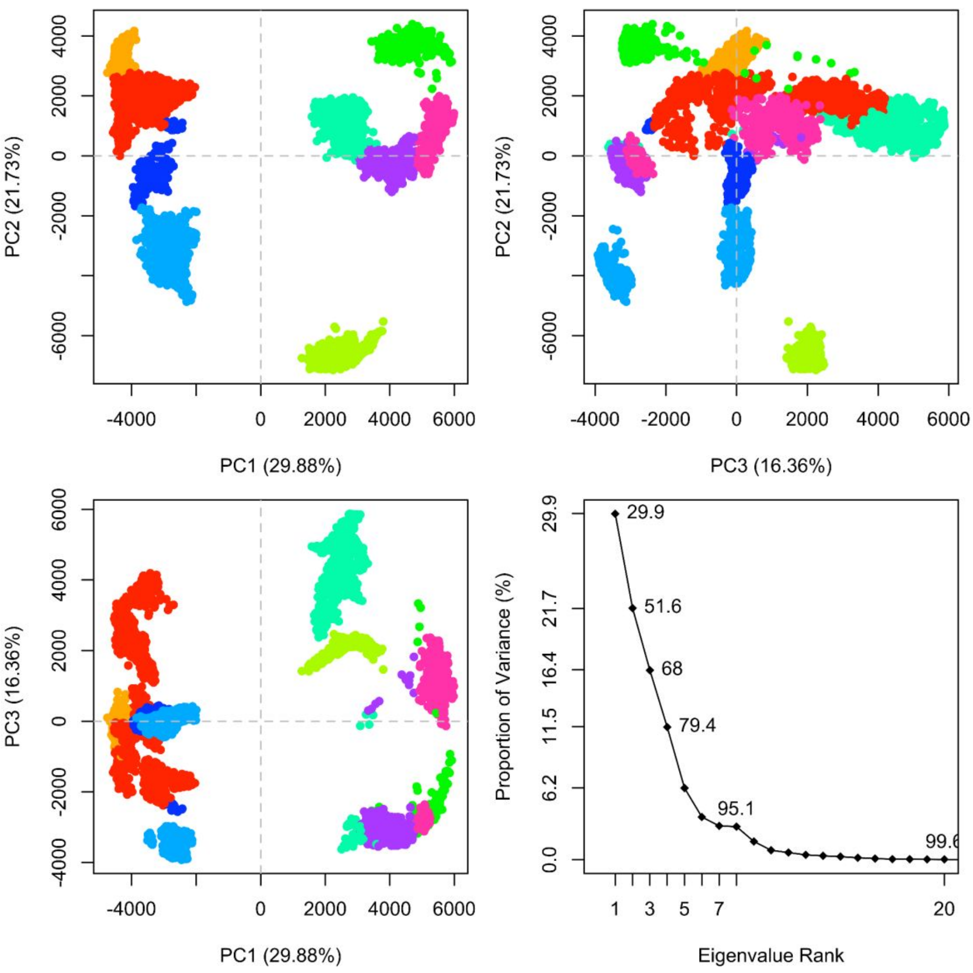

PC1 (29.88%)
PC2 (21.73%)
PC3 (16.36%)
Proportion of Variance (%)
Eigenvalue Rank
29.9
51.6
68
79.4
95.1
99.6

A

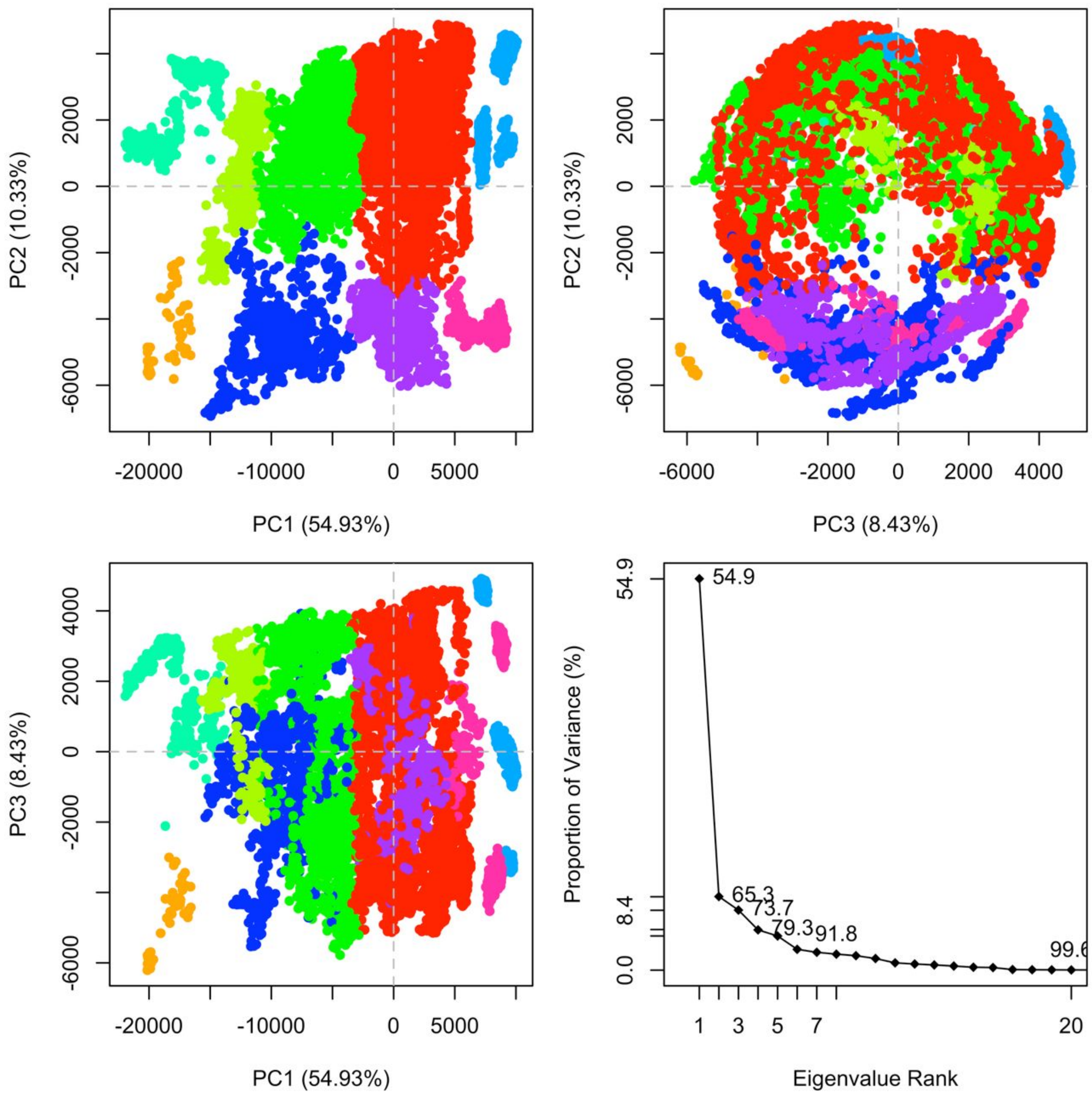

B

Figure S2. Principal Component Analysis (PCA) of two Fab molecular dynamics simulations at (A) pH 3.5, 0 mM NaCl, 338 K, and (B) pH 7, 50 mM NaCl, 338 K. The scatter plots show clustering of conformations based on the first three principal components: PC1 vs PC2 (top left), PC2 vs PC3 (top right), and PC1 vs PC3 (bottom left). Each colour represents a distinct cluster identified during analysis. The scree plot (bottom right) shows the cumulative proportion of variance explained by the top 20 eigenvalues, with PC1, PC2, and PC3 together accounting for approximately 68% (in A) and 73.7% (in B) of the total variance, respectively.

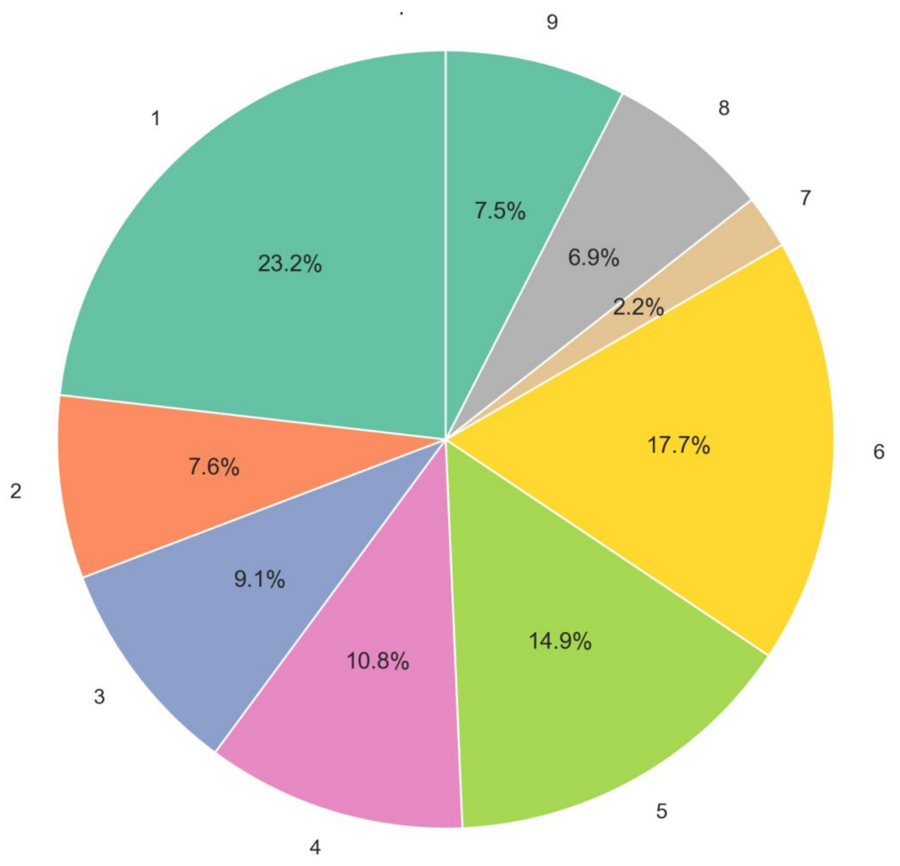

A

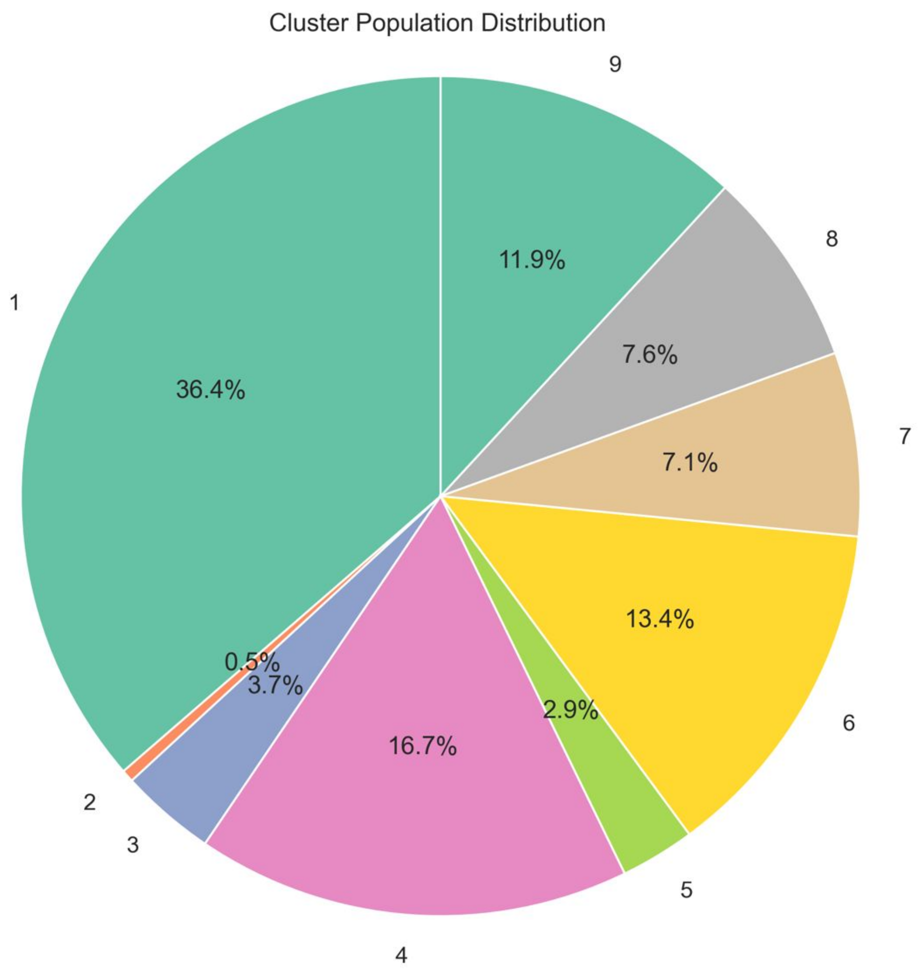

B

Figure S3. Population distribution of the nine clusters identified by principal component analysis (PCA) at (A) pH 3.5, 0 mM NaCl, 338 K, and (B) pH 7, 50 mM NaCl, 338 K.

Table S1. Selected hotspot regions based on frequency contact occurrence. The highlighted areas (yellow and blue) are the seven selected regions for their high occurrences in both Fabs in the simulations at pH 3.5, 0 mM NaCl, 338 K. Only one additional significant hotspot was observed in the pH 7, 50 mM NaCl, 338K simulations, at residues 187-190.

Residue index	Fab 1	Fab 2
26	29	221
27	192	973
28	575	1189
68	402	113
126	631	43
127	377	48
128	226	51
152	295	28
153	334	26
201	14	21
202	36	26
203	57	12
270	86	508
288	318	186
289	225	296
290	144	323
291	33	322
346	39	233
348	13	425
349	453	327
350	749	379
351	728	281

352	472	772
376	837	323
405	292	95
406	86	37
407	68	213
408	354	308
409	835	403
410	901	105
411	655	40
431	181	67
432	231	65
433	184	284
436	39	69
437	648	373
438	802	715
439	132	1698
440	641	1934
441	215	1457
442	435	1965

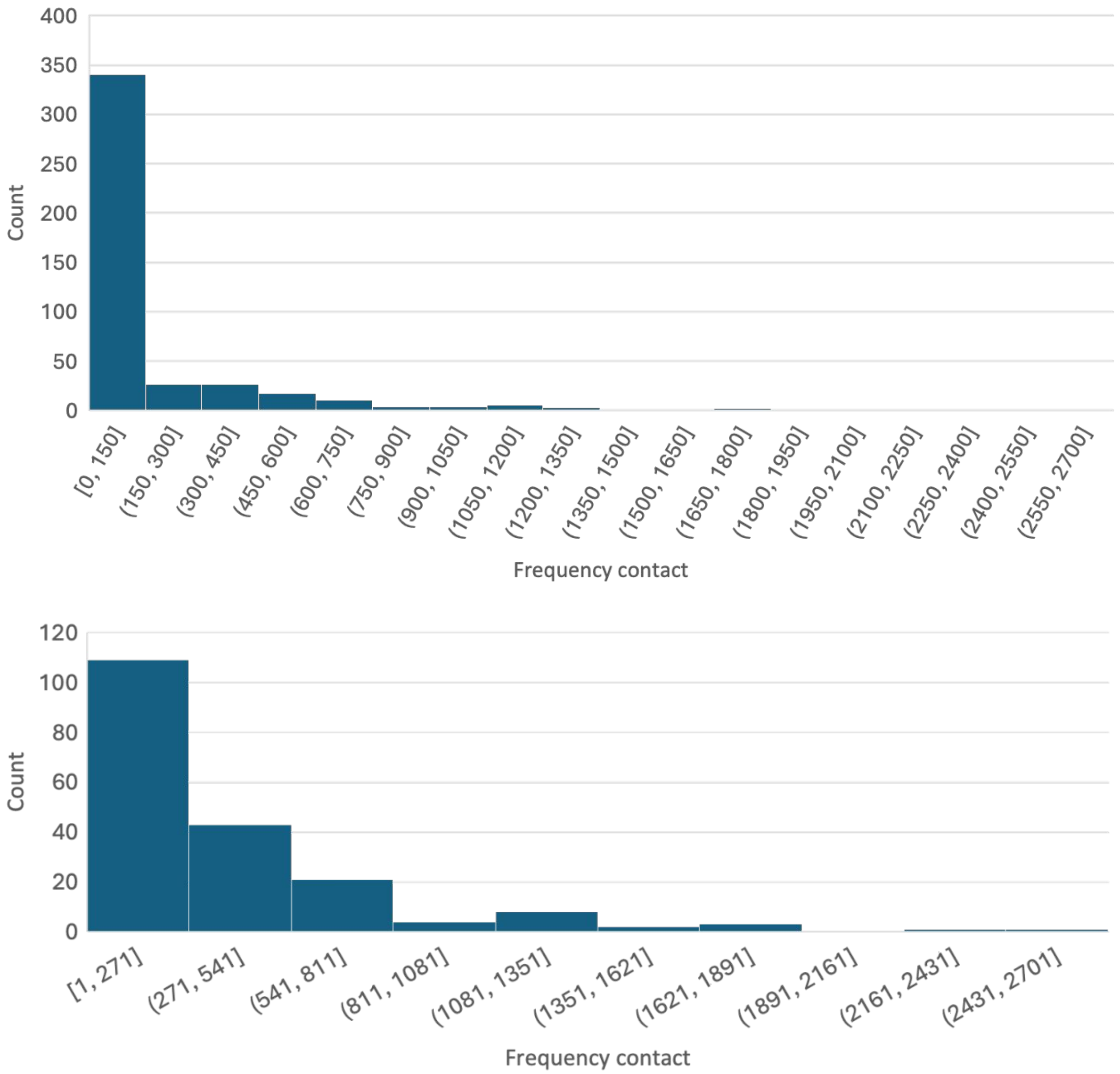

Figure S4. Histogram showing the distribution of contact frequencies between the two Fab fragments across all simulations and starting positions at pH 3.5, 0 mM NaCl, 338 K. TOP: all residues; BOTTOM: residues with zero contacts across all frames removed.

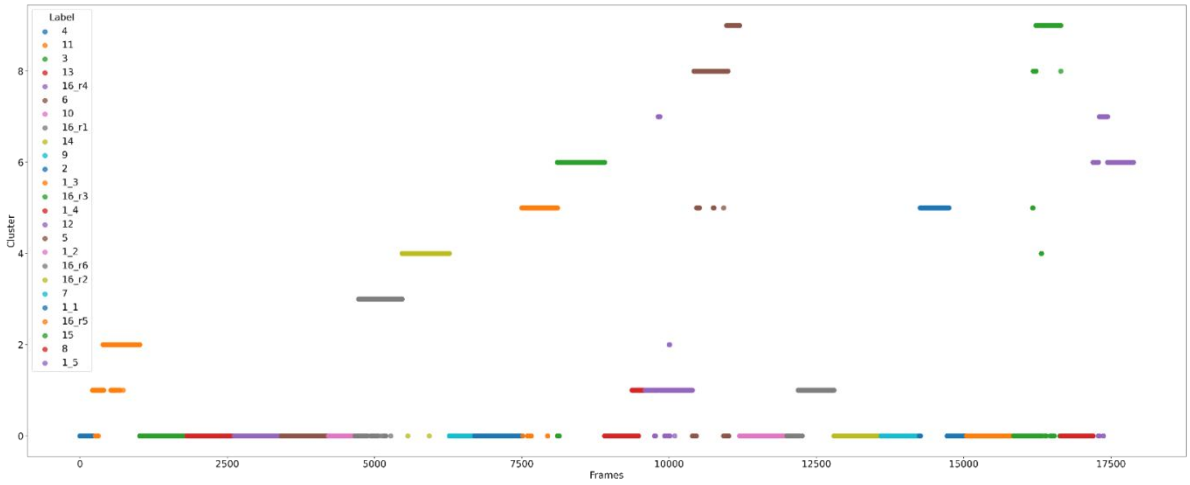

Figure S5. Occupancy of the nine PCA Clusters over time across the 16 MD simulations at pH 3.5, 0 mM NaCl, 338 K

Each dot represents a frame assigned to one of 9 principal component (PCA) clusters. Colours indicate the MD simulation trajectory label. PCA was performed on the final ~720 frames of each 100 ns simulation to exclude early-stage equilibration effects. The first 100 ns of each trajectory is therefore not plotted above, and all trajectories began in the cluster designated “0”, i.e. with no protein-protein interactions present.

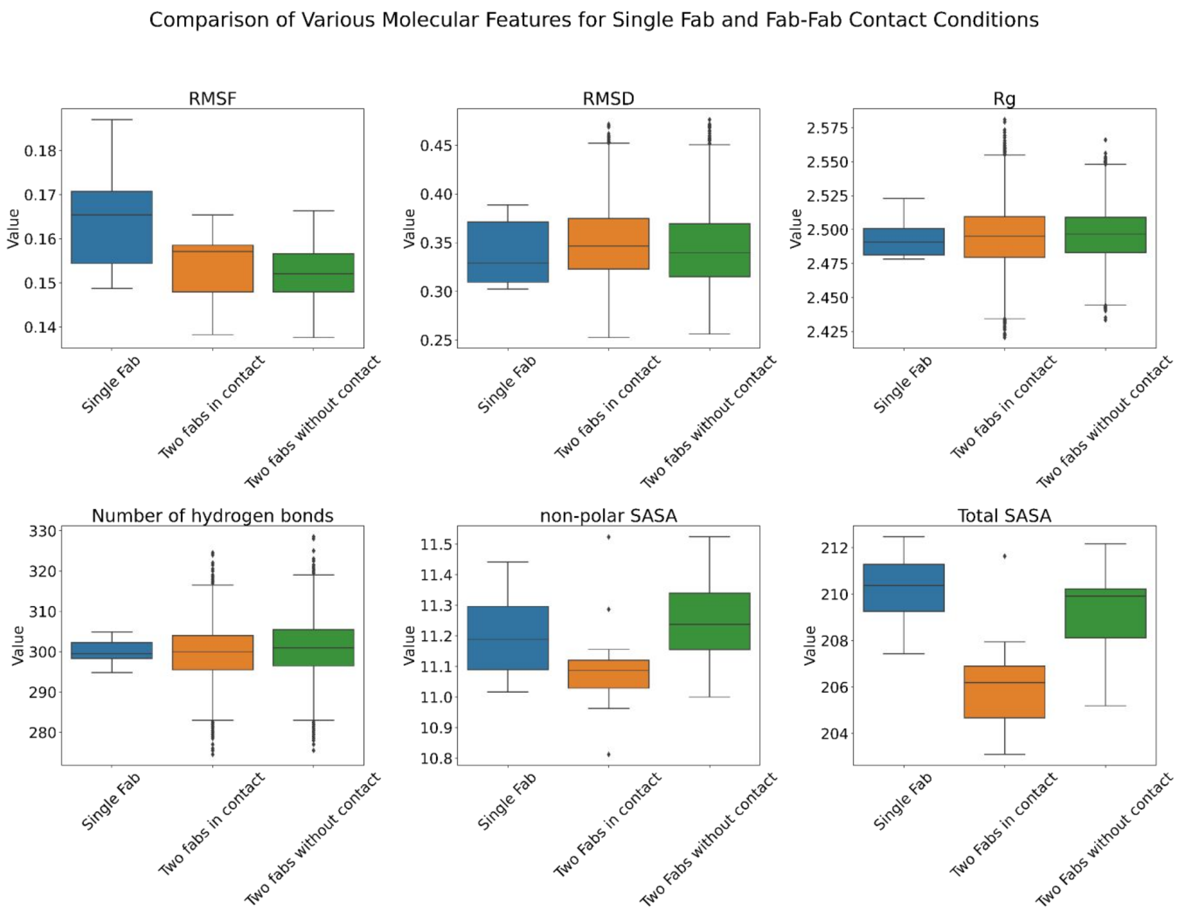

Figure S6. Comparison of various molecular features between single Fab and two Fab simulations under contact and non-contact conditions at pH 3.5, 0 mM NaCl, 338 K. Metrics shown include RMSF, RMSD, radius of gyration (R_g), number of hydrogen bonds, non-polar solvent-accessible surface area (SASA), and total SASA. Each box plot represents the distribution of values across simulation replicas. Error bars are the 95% confidence interval, the bottom and top of the box are the 25th and 75th percentiles, the line inside the box is the 50th percentile (median), and any outliers are shown as black dots.

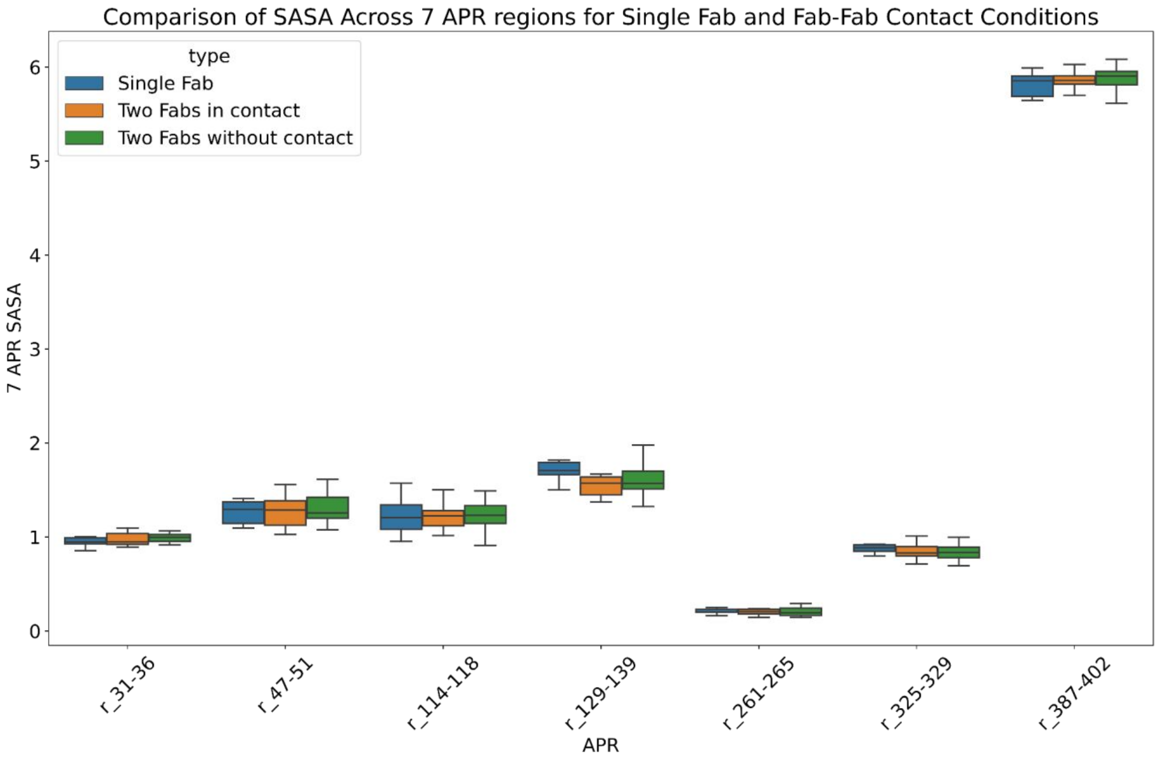

Figure S7. Comparison of solvent-accessible surface area (SASA) across the seven aggregation-prone regions (APRs) between the single Fab and two-Fab simulations at pH 3.5, 0 mM NaCl, 338 K.

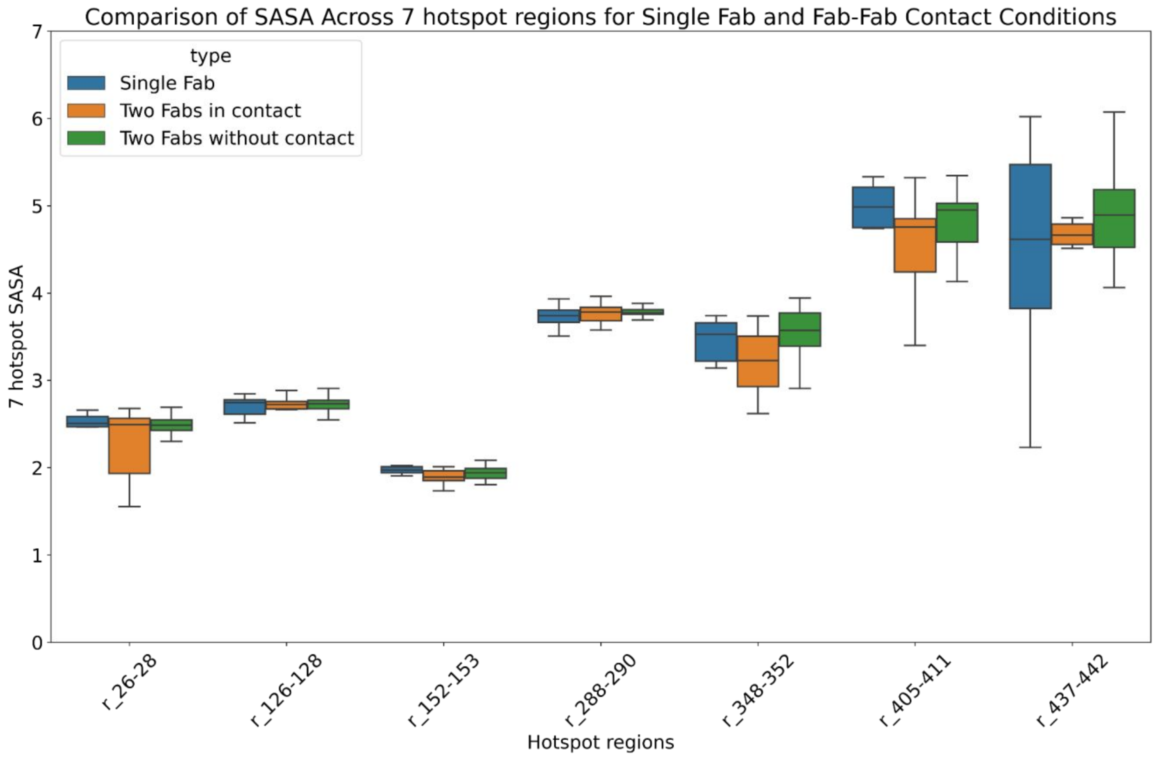

Figure S8. Comparison of solvent-accessible surface area (SASA) across the seven hotspot regions between the single Fab and two-Fab simulations at pH 3.5, 0 mM NaCl, 338 K.

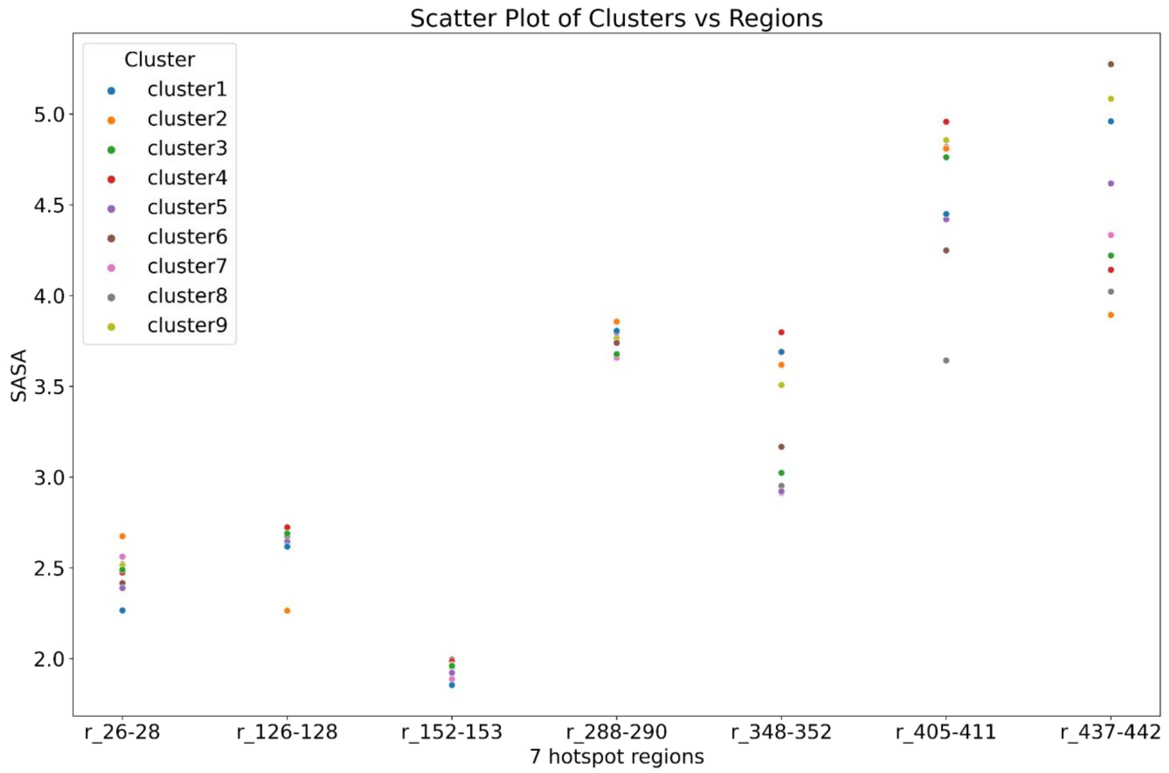

Figure S9. Scatter plot showing the average solvent-accessible surface area (SASA) of seven hotspot regions across nine clusters identified by principal component analysis (PCA) at pH 3.5, 0 mM NaCl, 338 K. Each point represents a hotspot's average SASA in a specific cluster.

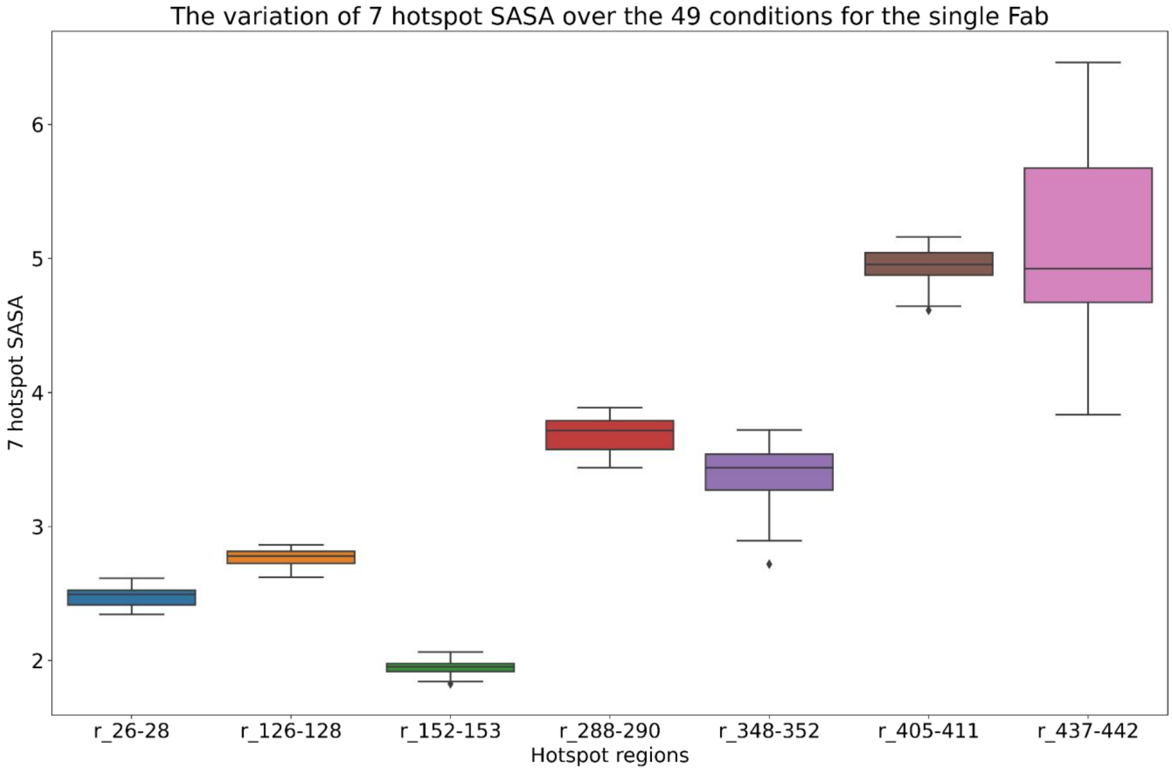

Figure S10. Boxplot showing the distribution of average solvent-accessible surface area (SASA) values for the seven identified hotspot regions, across all 49 simulation conditions for the single Fab. Each box represents a specific hotspot, and the variability in SASA suggests differing exposure profiles among hotspots, potentially contributing differently to Fab–Fab interaction interfaces.

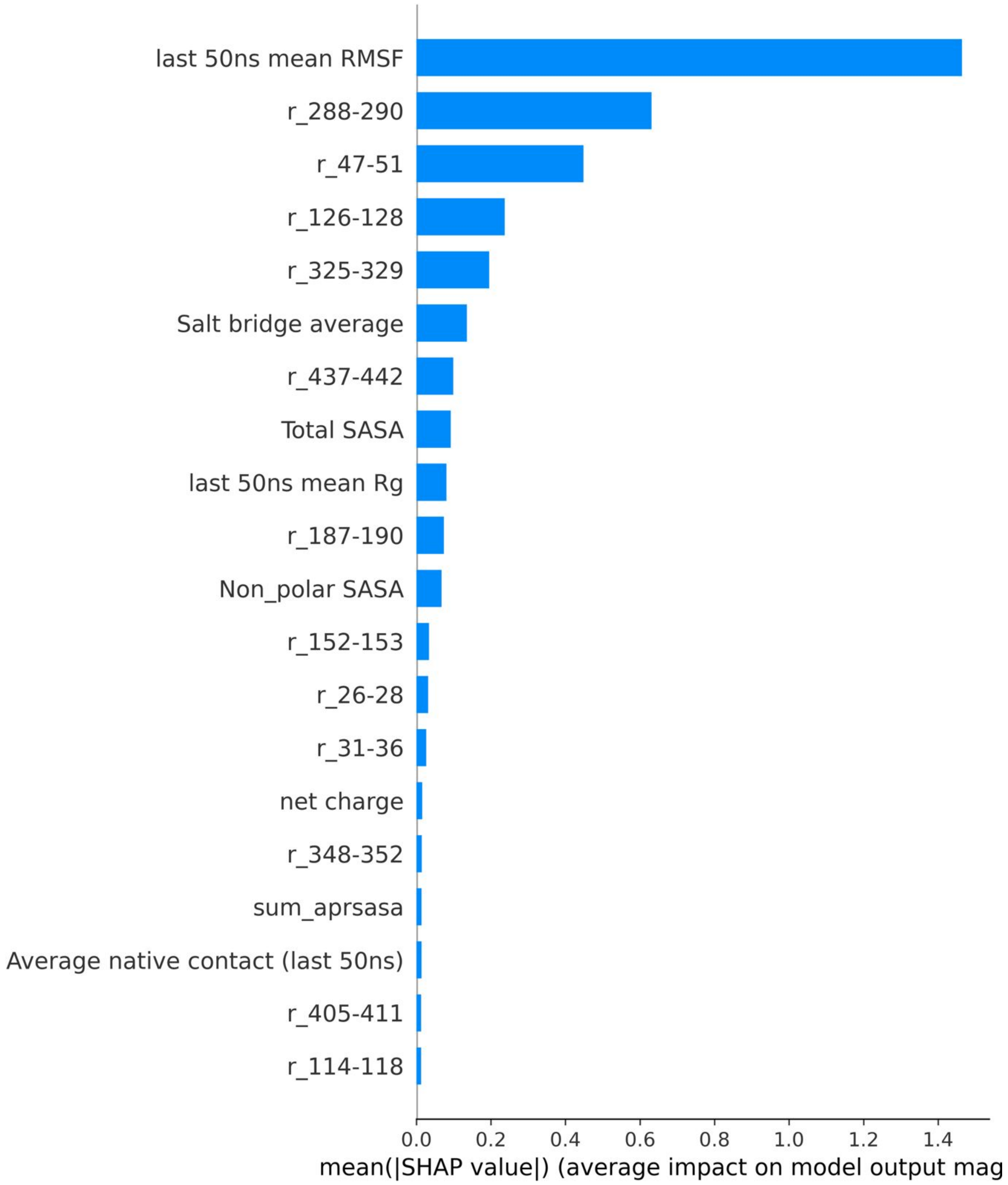

Figure S11. **SHAP analysis showing the relative contributions of all the molecular features to the XGBoost model.** The bar chart represents the average impact of each feature on the model's output, with longer bars indicating higher feature importance. This indicated the extra hotspot region identified only at pH 7 (residues 187-190) was not important enough to be included in the model building.
